# LncRNA MALAT1 promotes development of mantle cell lymphoma by associating with EZH2

**DOI:** 10.1186/s12967-016-1100-9

**Published:** 2016-12-20

**Authors:** Xin Wang, Lalit Sehgal, Neeraj Jain, Tamer Khashab, Rohit Mathur, Felipe Samaniego

**Affiliations:** 1 Department of Hematology, The First Affiliated Hospital of Chongqing Medical University, Chongqing, 400016 China; 2 Department of Lymphoma and Myeloma, The University of Texas MD Anderson Cancer Center, 1515 Holcombe Blvd., Houston, TX 77030 USA; 3 Department of Internal Medicine, Lankenau Medical Center, Wynnewood, PA USA

**Keywords:** Long non-coding RNA, MALAT1, MCL, Cell cycle, EZH2, Phosphorylation

## Abstract

**Background:**

Mantle cell lymphoma (MCL) is considered an aggressive subtype of non-Hodgkin’s lymphoma with variable treatment responses. There is an urgent need to identify novel markers with prognostic and therapeutic value for MCL. Long non-coding RNAs (lncRNAs) have emerged as key regulators in cancers, including MCL. Metastasis-associated lung adenocarcinoma transcript 1(MALAT1), a lncRNA located at pathognomonic translocation site of t (11; 14) of MCL. MALAT1 is known to be overexpressed in solid tumors and hematologic malignancies. However, the pathological role and clinical relevance of MALAT1 in MCL are not completely understood.

**Methods:**

We quantified MALAT1 in MCL samples (40) and CD19+ B cells by quantitative real time polymerase chain reaction (qRT-PCR) and correlated levels with clinical outcome. We silenced MALAT1 in MCL cell lines and analyzed cells in tumorigenic assays and formation of transcription complexes.

**Results:**

We found that the expression of MALAT1 was elevated in human MCL tumors and cell lines as compared to normal controls, and the elevated levels of MALAT1 correlated with higher MCL international prognostic index (MIPI) and reduced overall survival. MCL with knockdown of MALAT1 showed impaired cell proliferation, facilitated apoptosis and produced fewer clonogenic foci. The increased expression of p21 and p27 upon MALAT1 knockdown was regulated by enhancer of zeste homolog 2 (EZH2). Moreover, decreased phosphorylation of EZH2 at T350 attenuated the binding to MALAT1.

**Conclusions:**

Our findings illuminate the oncogenic role of MALAT1, which may serve as a novel biomarker and as a therapeutic target in MCL.

**Electronic supplementary material:**

The online version of this article (doi:10.1186/s12967-016-1100-9) contains supplementary material, which is available to authorized users.

## Background

With an annual incidence of 0.5 per 100,000 populations in Western countries, mantle cell lymphoma (MCL) is an aggressive subtype of non-Hodgkin’s lymphoma (NHL), comprising about 6% of NHL cases [1]. Despite an initial therapeutic response, patients consistently develop recurrence and chemoresistance [2]. Moreover, elderly patients with MCL do not tolerate the toxicities of chemotherapy. Thus, there is a substantial need to identify novel markers for prognosis and explore alternative therapies for MCL patients.

Over half of the human genome is actively transcribed as noncoding RNAs [3]. The noncoding transcripts that are more than 200 nucleotides in length are termed long noncoding RNAs (lncRNAs). lncRNAs have been recognized to play important roles in pathologic conditions, such as cancer [4, 5]. It has been demonstrated that lncRNAs influence tumor progression through modulating cell cycle, survival, immune response or pluripotency, through their interactions with DNA, protein, and RNA [6, 7].

Recent studies in lymphoma have shown that lncRNA FAS-AS1 regulates skipping of exon 6 through direct interaction with RBM5, in order to produce membrane associated Fas or decoy soluble Fas [8]. In addition, expression of lncRNAs (PEG10, LUNAR1 and HULC) is correlated with clinical poor prognosis in diffuse large B cell lymphoma (DLBCL). Reduced expression of these same lncRNAs inhibited cell proliferation of DLBCL in vitro [9–11]. Additional lncRNAs have been discovered to regulate oncogenic process in lymphomas. For example, Verma Asyu et al. examined RNA-seq data from primary DLBCL tumors and identified 2632 novel lncRNAs, which are implicated to interact with EZH2, H3K4me3, NFκB and STAT3 [12]. Another study using microarray analysis reported 189 lncRNAs that were significantly dysregulated in follicular lymphoma (FL), which are related to the TNF signaling and virus related carcinogenesis [13].

Metastasis-associated lung adenocarcinoma transcript 1(MALAT1) is an evolutionarily conserved, long non-coding RNA 8.7 kb transcript, located on chromosome 11q13, a site in vicinity of t (11;14) of MCL. Chromosomal translocations are known to influence expression of local genes. MALAT1 plays a critical role in maintaining the proliferation potential of early-stage hematopoietic cells [14]. MALAT1 is differentially regulated during the activation of B-cells, and recent reports implicate it as a target of activation-induced deaminase (AID), which can induce oncogenic translocations in germinal center B-cells [15]. The expression level of MALAT1 is correlated with tumorigenesis and metastasis in solid cancers and multiple myeloma, suggesting a universal cancer role [16]. Despite many previous studies, the role of MALAT1 in lymphoma is not yet fully understood. In this study, we will analyze the role of MALAT1 in the pathophysiologic process of MCL.

## Methods

### Patient samples

Mantle cell lymphoma patients’ samples and clinical information were collected and published under The University of Texas MD Anderson Cancer Center IRB-approved clinical protocol LAB08-0190 for use of human tissues, with the written informed consent of all patients. Information about the patients is shown in Additional file 1: Table S1. Normal B lymphocytes were isolated from peripheral blood of healthy donors’ blood, obtained from Gulf Coast Blood Center (Houston, TX, USA), with CD19+ magnetic beads and released with DETACHaBEAD CD19 (Invitrogen, Grand Island, NY, USA).

### Cell culture

Mantle cell lymphoma cell lines (Mino and Jeko-1) were obtained from ATCC (Manassas, VA, USA). Cell lines were regularly tested for mycoplasma (Lonza MycoAlert) and authenticated at the Cell Line Core Facility at MD Anderson Cancer Center, University of Texas, Houston, TX, USA. The cell lines were cultured in RPMI 1640 medium with 10% fetal bovine serum (FBS, Gibco). The human cell lines were validated based on short tandem repeats (STR). STR repeats are regions of microsatellite instability with defined tri- or tetrad-nucleotide repeats that are located throughout the chromosomes. PCR reactions using primers on non-repetitive flanking regions generate PCR products of different sizes based on the number of repeats in the region; the size of these PCR products are determined by capillary electrophoresis. This is performed by core facility at MD Anderson cancer center.

### RNA isolation and quantitative real-time PCR

Total RNA was extracted from patients MCL tissue or cell lines using RNeasy kit (QIAGEN) according to the manufacturer’s instructions. Reverse transcription reactions were performed using a SuperScript III reverse transcriptase kit (Invitrogen-Life Technologies) according to the manufacturer’s protocol. Quantitative real-time RT-PCR was performed in triplicate using the StepOnePlus Real-Time PCR System (Applied Biosystems-Life Technologies) with TaqMan Universal PCR Master Mix according to the manufacture’s protocol (Applied Biosystems). The TaqMan Gene Expression Assays (probe and primers) were purchased from Invitrogen Life Technologies, including MALAT1 (ID: Hs00273907), EZH2 (ID: Hs00544833), CDKN1A/p21 (ID: Hs00355782) and CDKN1B/p27 (ID: Hs01597588). Human GAPDH was used as endogenous control. StepOne software version 2.0 (Applied Biosystems) was used to determine RNA expression levels.

### RNA interference

Mantle cell lymphoma cell lines were transfected with Neo transfection (Invitrogen) using five human MALAT1 siRNAs (si-MALAT1, Life Technologies): si-MALAT1 No.1 (product ID: 272231), si-MALAT1 No.2 (product ID:272233), si-MALAT1 No.3 (product ID:272234), si-MALAT1 235 (product ID:272235) and si-MALAT1 236 (product ID:272236), or negative control siRNA (si-NC, product ID: AM4635; Life Technologies). Transfection was performed three times to confirm results. Briefly, MCL cells were resuspended in Buffer R (Invitrogen) and mixed with a siRNA. Each 100 μL aliquot contained 2 × 10^6^ cells and 1 nmol of siRNA. After electroporation with program parameters (1550 V, 10 ms, 3 pulses) cells were cultured in RPMI 1640 and 10% FBS without antibodies. Quantitative real-time PCR was performed to evaluate expression levels of MALAT1 using total RNA extracted 48 h after transfection (si-NC, si-MALAT) in Mino and Jeko-1 cells.

### Evaluation of proliferation, apoptosis and colony formation assay

Cell viability was measured 24, 48 and 72 h after transfection with MTT assay kit (Promega, Madison, WI). Flow cytometry analysis for apoptosis was performed using Annexin V-FITC Apoptosis Detection Kit 48 h after transfection according to the manufacturer’s protocol (Sigma–Aldrich). All experiments were performed in triplicate. The colony formation assay was performed as described previously [17]. In brief, 5 × 10^4^ cells were mixed with methylcellulose (H4100; Stemcell Technologies, Vancouver, BC, Canada) containing RPMI-1640 + 10% FBS and poured in 35 mm plate. The colonies were allowed to grow for 7–14 days, followed by staining with p-Iodonitrotetrazolium violet and counted using Fluorchem 8800 imaging system (Alpha-Innotech, San Leandro, CA).

### Cell synchronization and cell cycle analysis

Mino and Jeko-1 cells were synchronized to G2/M phase by treatment with nocodazole (Selleckchem). To synchronize cells in G1, the synchronized G2/M cells were washed with PBS and grown in fresh medium for 12 h. Briefly, Mino and Jeko-1 cells were collected 24 h after transfection, and treated with 50 ng/ml nocodazole for 24 h, then released in fresh medium. Cells were collected at 12 and 18 h after release and processed to ethanol fixing (70% ethanol, ice-cold), RNase A-pretreating (0.5 mg/ml at 37 °C for 30 min) and propidium iodide staining (50 μg/ml). Cell-cycle progression was measured by flow cytometry.

### RNA immunoprecipitation assay

RNA immunoprecipitation (RIP) was performed using a Magna RIP RNA-Binding Protein Immunoprecipitation Kit (Millipore) according to the manufacturer’s instructions. Antibodies EZH2 (#5246; Cell Signaling Technology) and SUZ12 (#3737; Cell Signaling Technology) used in RIP assays were diluted as 1:100. The coprecipitated RNAs were transcribed to cDNA, and detected by quantitative real-time RT-PCR using TaqMan Gene Expression Assays (Invitrogen Life Technologies).

### Chromatin immunoprecipitation assay

Chromatin immunoprecipitation (ChIP) was performed using the EZ-ChIP Chromatin Immunoprecipitation Kit (Millipore) according to the manufacturer’s instruction. Briefly, cross-linked chromatin was sonicated on a Branson Sonifier 150 at setting 4 with 15 s pulses six times on ice. Then, the chromatin was immunoprecipitated using anti-EZH2 (#5246; Cell Signaling Technology) or anti-H3K27me3antibodies (#17-622, EMD Millipore). The quantitative real-time PCR was used to detect immunoprecipitated DNA using SYBR Green PCR Master Mix (Applied Biosystems) according to the method described above. Primers were used to identify promoter domain in immunoprecipitated DNA: CDKN1A/p21 (5′-TCTGGGGTCTCACTTCTTGG-3′; 5′-ATGTGAGGAAGGCTCAGTGG-3′) and CDKN1B/p27 (5′-GATGGGGTTCACCGTGTTAG-3′; 5′-CCCTTTCCAAACATCCATTG-3′).

### Western blot analysis

Western blot analysis was performed as previously described [8, 18]. The antibodies used were specific for p21 Waf1/Cip1 (#2947, Cell Signaling Technology), p27 Kip1 (#2552, Cell Signaling Technology), EZH2 (#5246; Cell Signaling Technology), H3K27me3 (#39155, Active Motif), Histone H3 (#9715, Cell Signaling Technology) and pEZH2 T345 (#61241, Active Motif, recognize human pEZH2 T350). Anti-β-actin horseradish peroxidase antibody (1:10,000; Sigma Aldrich, Buchs, Switzerland) was used as loading control. Visualization was achieved by Supersignal West Pico chemiluminescent (or Femto Maximum Sensitivity) substrate (Pierce).

### Statistical analysis

Experimental data are presented as mean ± SD from three independent experiments, unless otherwise indicated. Differences between groups were calculated using the two-tailed Student’s *t* test (GraphPad Prism, La Jolla, CA, USA). Correlation between MALAT1 mRNA and EZH2 mRNA expression in human MCL tissues was examined with two-sided Pearson correlation. Overall survival was estimated with Kaplan–Meier method. P < 0.05 was considered statistically significant.

## Results

### Clinical correlation between MALAT1 expression and overall survival in MCL patients

**Fig. 1:**
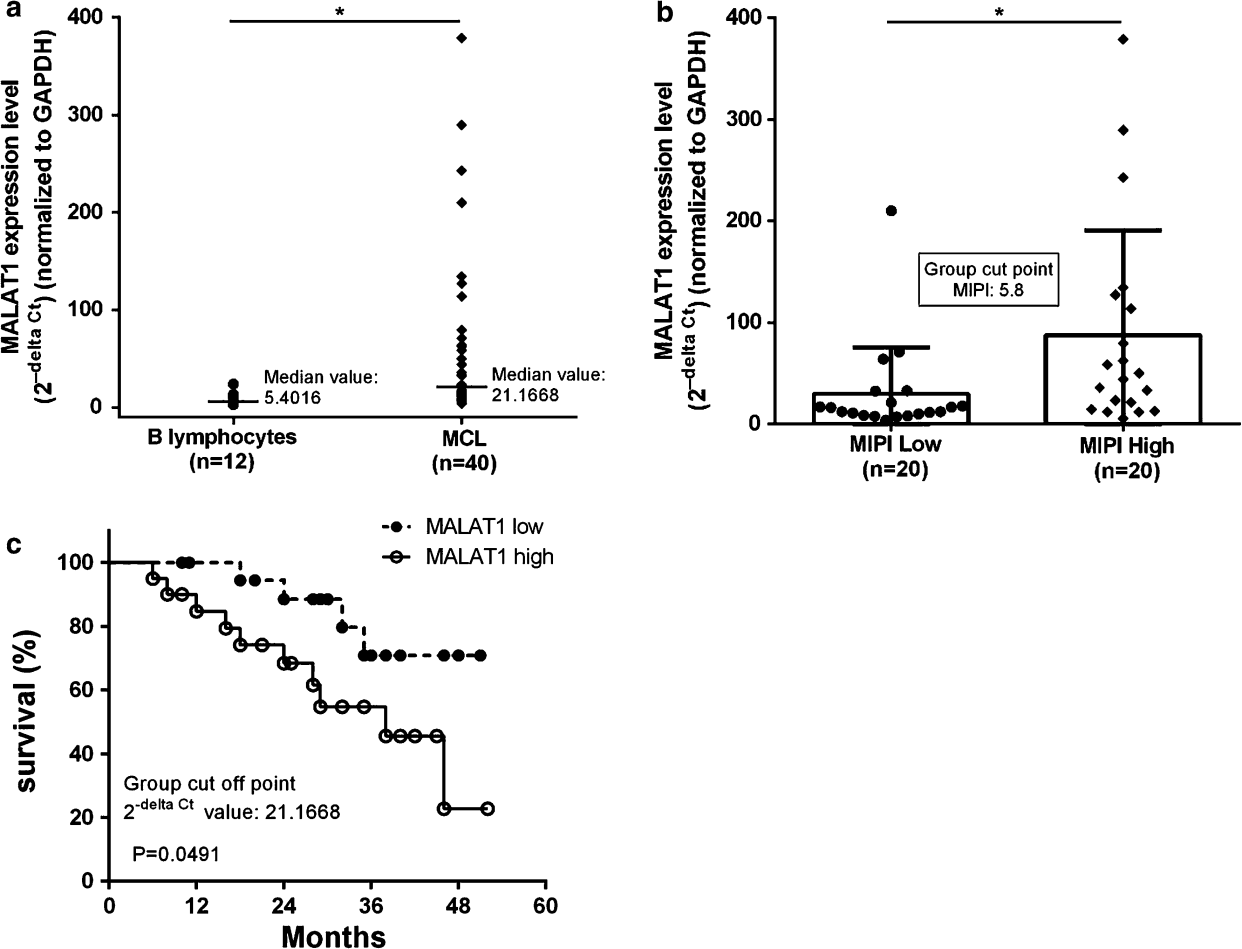
MALAT1 was over-expressed in MCL and is associated with clinical parameters. **a** Relative MALAT1 expression in primary human MCL tissues and CD19 + B lymphocyte from health donors, measured by qRT-PCR and normalized to gene expression levels of GAPDH. **b** The expression of MALAT1 was significantly higher in patients with high MIPI. **c** Kaplan–Meier plot of patients according to MALAT1 expression. Patients with high MALAT1 expression levels (n = 20) had a significantly lower overall survival than those with low expression (n = 20). Hazard ratio: 3.006 (95% CI: 1.012 to 8.299) Data represent the mean ± SD. *P < 0.05

First, we quantified MALAT1 expression level in 40 MCL samples and peripheral CD19+ B lymphocytes from 12 healthy donors by quantitative real time polymerase chain reaction (qRT-PCR), and the average of MALAT1 levels in the healthy donors was normalized to 1. MALAT1 expression was significantly higher in MCL tissue compared to healthy donor CD19 + B lymphocytes (P < 0.05; Fig. 1a). The Mantle Cell Lymphoma International Prognostic Index (MIPI) has been recently generated as a prognostic tool. Its scoring is based on a model using four clinical variables [19]. The 40 MCL patients were divided into two groups using the median MIPI score of 5.8 as cutoff. We found that the MALAT1 expression level were closely associated with MIPI (P < 0.05; Fig. 1b), and significantly higher in the high and intermediate risk groups compared to the low risk group of MIPI (Additional file 1: Table S1) [19]. Next, we divided the MCL patients into two groups according to MALAT1 expression levels, using a median MALAT1 2^−ΔCt^ value of 21.1668. Then the association between the MALAT1 expression level and overall survival was assessed through Kaplan–Meier analysis and log-rank test. Results showed MALAT1 high expression group had significant shorter overall survival than MALAT1 low group (P < 0.05; Fig. 1c).

### Effect of MALAT1 on MCL cell proliferation, apoptosis, and cell cycle progression

**Fig. 2:**
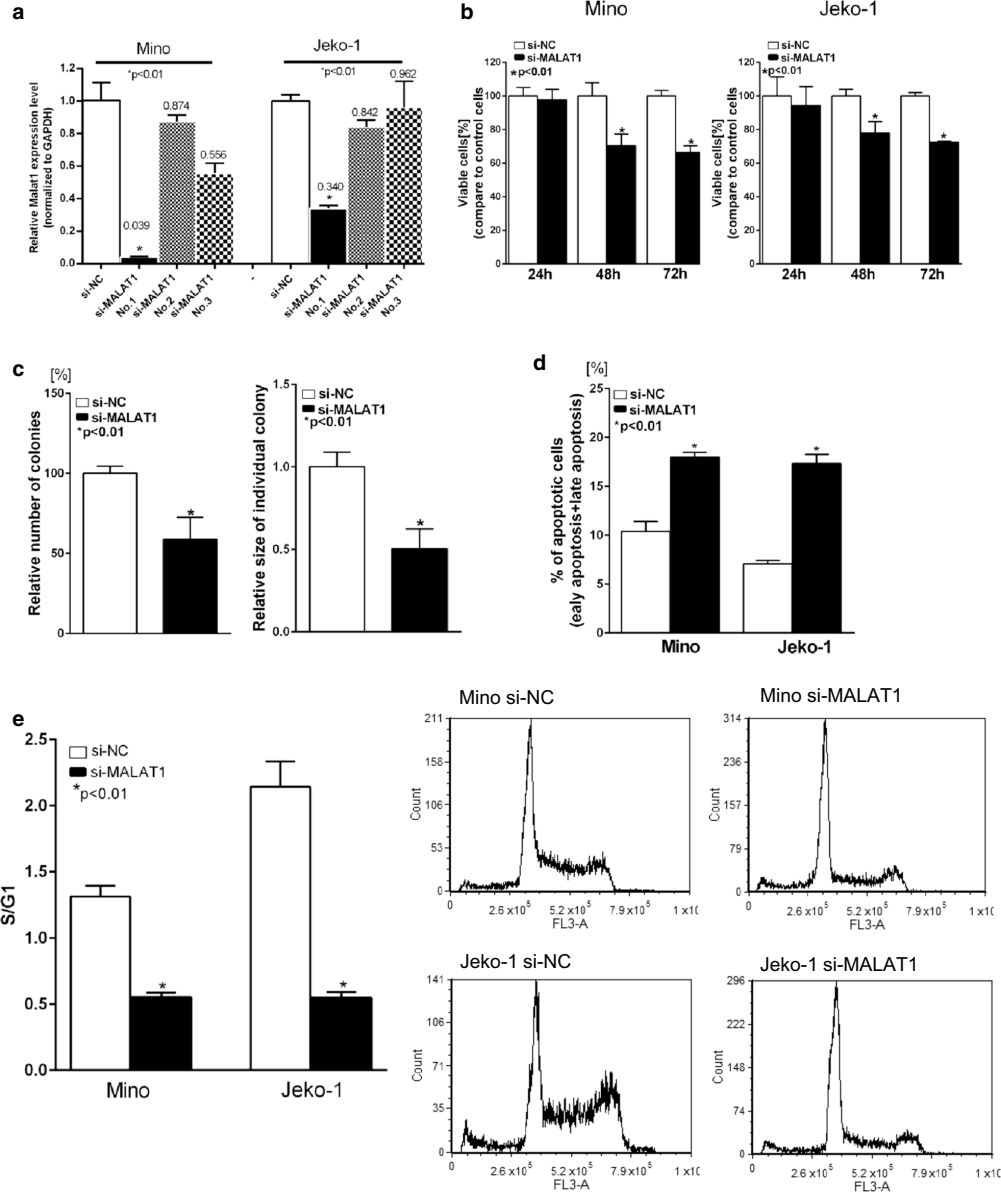
Knockdown of MALAT1 inhibited malignant potential of MCL. **a** Knockdown of MALAT1 with si-MALAT1(No.1, No.2 and No.3) and nontarget control analyzed by qRT-PCR. **b** Cell viability assay on MCL cells transfected with si-MALAT1 or si-NC. **c** Colony formation assay. The number of colonies significantly decreased, and the size of individual colonies were significantly reduced in MALAT1 knockdown Mino cells. **d** Apoptosis in MCL cells transfected with si-MALAT1 or si-NC was detected by flow cytometry after annexin V/PI staining. **e** Cell cycle analysis by flow cytometry. Ratio of percentage of cells in S phase to G1 phase was significantly decreased in MALAT1 knockdown MCL cells (Mino and Jeko-1). Data are representative of three independent experiments and represent the mean ± SD

The basal expression level of MALAT1 was determined in six mantle cell lymphoma derived cell lines (Z-138, Mino, REC-1, Jeko-1, JVM2 and Granta-519) (Additional file 2: Figure S1), the MCL cells with higher expression of MALAT1 (Mino and Jeko-1) which were used for additional experiments. Transient transfection was conducted with si-MALAT1 using electroporation on Mino and Jeko-1 cells. The knockdown effect was most profound using si-MALAT1 No.1 compared with si-MALAT1 No.2 and No.3, which caused 96.1 and 66% decrease of MALAT1 levels in Mino and Jeko-1 cells, respectively (Fig. 2a). si-MALAT1 236, decreased MALAT1 expression in Mino and Jeko-1 cells (Additional file 3: Figure S2a). Thus, si-MALAT1 No.1 and si-MALAT1236 were chosen for further experiments. To evaluate the effects of MALAT1 deletion on tumor cell proliferation, we used MTT and colony formation assays. As shown in Fig. 2, we observed significantly reduced cell viability after MALAT1 knockdown. Compared to the si-NC groups, the cell viability of Mino and Jeko-1 cells transfected with si-MALAT1 was decreased by 33.6 and 27.6% after 72 h of incubation, respectively (P < 0.01; Fig. 2b; Additional file 3: Figure S2b). In addition, we found that si-MALAT1 transfected Mino cells were less clonogenic in methylcellulose compared to si-NC cells (Additional file 4: Figure S3), and the size of individual colonies was also significantly smaller in si-MALAT1 MCL cells (P < 0.01; Fig. 2c; Additional file 4: Figure S3b). Moreover, the percentage of apoptotic cells was increased 48 h after transfection with si-MALAT1 in Mino and Jeko-1 cells, respectively (P < 0.01; Fig. 2d; Additional file 5: Figure S4, Additional file 6: Figure S5). Next, we explored the effect of MALAT1 on cell cycle progression. Mino and Jeko-1 cells could not be arrested in G0 by serum starvation, perhaps due to the absence of a quiescent state in MCL cells. Then, we synchronized Mino and Jeko-1 cells in G2/M phase with nocodazole and released them with or without MALAT1 knockdown at different time points (12 and 18 h after release) and examined the cell cycle progression (Additional file 7: Figure S6). We observed cell cycle arrest at the S/G1 transition in si-MALAT1 transfected MCL cells (Mino and Jeko-1) (Fig. 2e; Additional file 7: Figure S6b, Additional file 8: Figure S7).

### MALAT1 binding to polycomb repressive complex 2 (PRC2) through EZH2

**Fig. 3:**
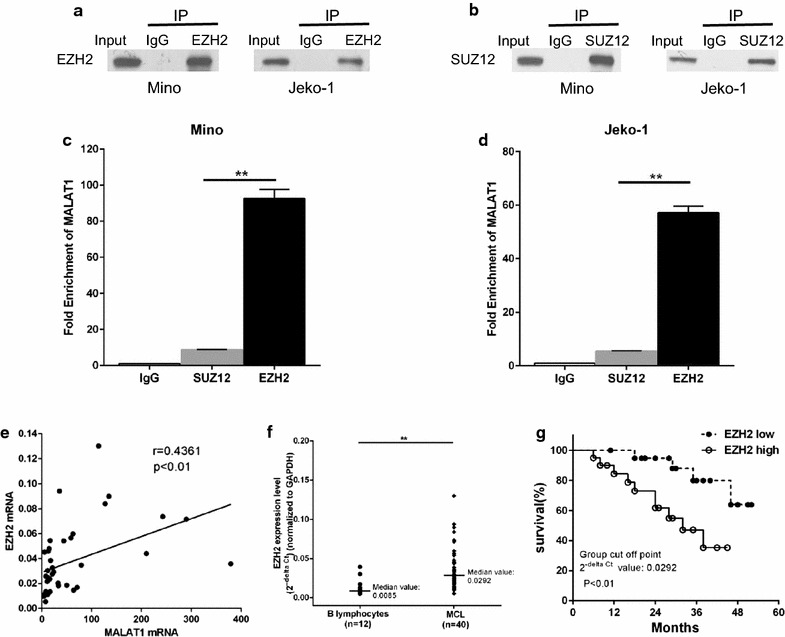
Correlation between MALAT1 and EZH2 in MCL. **a–d** RNA immunoprecipitation in Mino and Jeko-1 cells. **a** Input and immunoprecipitate from rabbit IgG and anti-EZH2 antibody bond beads, detected by immunoblotting with EZH2 antibody, **b** Input and immunoprecipitate from rabbit IgG and anti-SUZ12 antibody bound beads, detected by immunoblotting with SUZ12 antibody, using Western blot analysis. **c**, **d** qPCR showing MALAT1 is significantly enriched in the immunoprecipitate from the EZH2 antibody bound beads compared to SUZ12 and IgG (control antibody) in (**c**) Mino and (**d**) Jeko-1 cells. **e** Pearson’s correlation between MALAT1 mRNA and EZH2 mRNA expression (2^−delta Ct^ value) in MCL. **f** Relative MALAT1 expression in MCL clinical samples and CD19 + B lymphocyte from healthy donors, measured by qRT-PCR and normalized to gene expression levels of GAPDH. **g** Overall survival of MCL patients with high and low EZH2 expression (Kaplan–Meier plot). Patients with high EZH2 expression levels (n = 20) had significantly lower overall survival than those with low expression (n = 20). Hazard ratio: 3.977 (95% CI: 1.512 to 13.01). Data represent the mean ± SD.**P < 0.01

Previous studies have revealed that several lncRNAs, such as HOTAIR and Xist, bind to PRC2 enhancing tri-methylation of histone H3 on lysine 27 (i.e. H3K27me3), that results in epigenetic silencing of target gene [20]. It was reported that MALAT1 also affect gene expression by interacting with PRC2 through different subunits (EZH2, SUZ12) [21, 22]. To investigate whether there is an interaction between MALAT1 and PRC2 in MCL, we performed RIP with antibodies against EZH2 or SUZ12. It is apparent that MALAT1 was significantly enriched with the EZH2 antibody and not with SUZ12 antibody and IgG (control antibody) in two MCL cell lines; P < 0.001; Fig. 3a–d). The expression levels of MALAT1 and EZH2 were compared in MCL samples, and we observed that there was a notable positive correlation between these two groups (Pearson correlation, r = 0.4361, P < 0.01; Fig. 3e).

### Association of EZH2 with clinical parameters in MCL

In same cohort of patients, we found the expression of EZH2 was significantly elevated in MCL tissues compared with healthy donors (P < 0.01; Fig. 3f). To further investigate the prognostic value of EZH2 on MCL, patients were divided into two groups according to EZH2 expression levels, using a median EZH2 2^−ΔCt^ value of 0.0292 as cutoff. We found expression level of EZH2 was significantly higher in high and intermediate risk groups compare to low risk group classified according to MIPI (P < 0.01; Additional file 9: Table S2) [19], and overall survival was also significantly lower in the high EZH2 expression group compared with the low group (P < 0.01; Fig. 3g).

### MALAT1 represses the expression of PRC2-dependent target genes by associating with EZH2

**Fig. 4:**
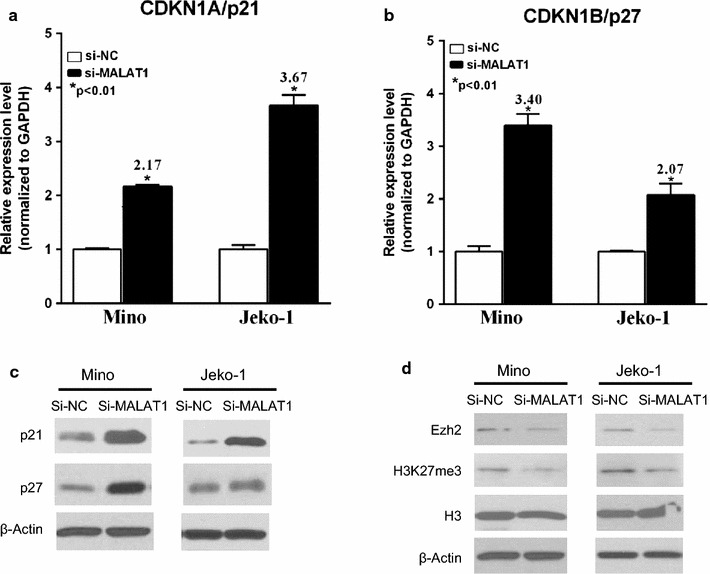
Effect of MALAT1 knockdown on the expression of EZH2 and H3k27me3, and cell cycle regulators p21 and p27. **a**, **b** qRT-PCR results for expressions of CDKN1A/p21 and CDKN1B/p27 (si-NC vs si-MALAT1). **c**, **d** The expression of EZH2 and H3k27me3 were suppressed, while p21 and p27 were increased in MALAT1 knockdown cells by *Western blot* analysis

It was reported that TUG1 was associated with PRC2, resulting in the epigenetic repression of cyclin-dependent protein kinase inhibitors, including p21 and p27, thus contributing to the regulation of cell cycle and proliferation in germinal center B (GCB) cells [23]. CDKN1A/p21 and CDKN1B/p27 have also been reported to be EZH2 target genes in lymphoma cell line SUDHL4, and suppressed by H3K27-trimethylation [24]. To investigate whether expression of the CDKN1A/p21 and CDKN1B/p27 were affected by MALAT1 in MCL, we analyzed p21and p27 expression after MALAT1 knockdown. As expected, the mRNA level of CDKN1A/p21 (P < 0.01; Fig. 4a) and CDKN1B/p27 (P < 0.01; Fig. 4b) were significantly increased in si-MALAT1 transfected MCL cell lines Mino and Jeko-1. A higher protein level of p21 and p27 in MALAT1 knockdown cells was observed (Fig. 4c; Additional file 10: Figure S8). Meanwhile, expression of EZH2 and H3K27me3 protein was decreased after MALAT1 knockdown in MCL cell lines (Fig. 4d; Additional file 10: Figure S8).

**Fig. 5:**
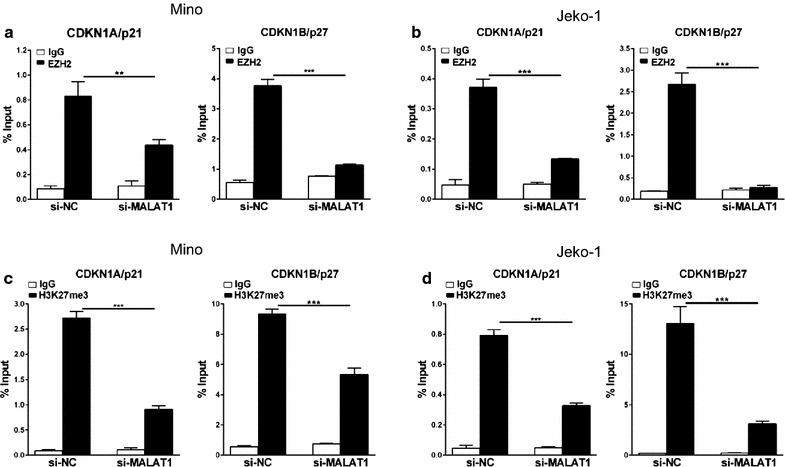
P21 and p27 expression were repressed by MALAT1 associating with EZH2. **a**, **b** ChIP analysis of Mino (**a**) and Jeko-1 (**b**) cells transfected with si-NC and si-MALAT1 were conducted on CDKN1A/p21 and CDKN1B/p27 promoter regions using anti-EZH2 antibody. **c**, **d** ChIP-qPCR analysis of H3k27me3 levels at the promoters of CDKN1A/p21 and CDKN1B/p27 using si-NC and si-MALAT1 transfected cells, Mino **(c**) and Jeko-1 (**d**). Data are mean ± S.D. from experiments with three replicates. **P < 0.01, ***P < 0.001

Our data shows that EZH2 binds directly to MALAT1 and enhances EZH2-mediated H3K27me3 and gene repression. To further determine whether MALAT1 increases EZH2 recruitment and H3K27me3 levels at EZH2 target loci, we performed a ChIP assay using anti-EZH2 and anti-H3K27me3 antibodies in MCL cells treated with si-MALAT1 or si-NC. The ChIP assay demonstrated that MALAT1 knockdown decreased EZH2 recruitment to the promoters of target genes CDKN1A/p21 and CDKN1B/p27 in Mino and Jeko-1 cells (Fig. 5a, b). Meanwhile, similar results were observed when we determined the levels of H3K27me3 at these gene promoters in both cell lines (Fig. 5c, d). These results indicate that MALAT1 represses expression of CDKN1A/p21 and CDKN1B/p27 by recruiting EZH2 and enhancing the H3K27me3 activity.

### EZH2 binding with MALAT1 can be self-enhanced by associating with p21/p27 and phosphorylation of EZH2

**Fig. 6:**
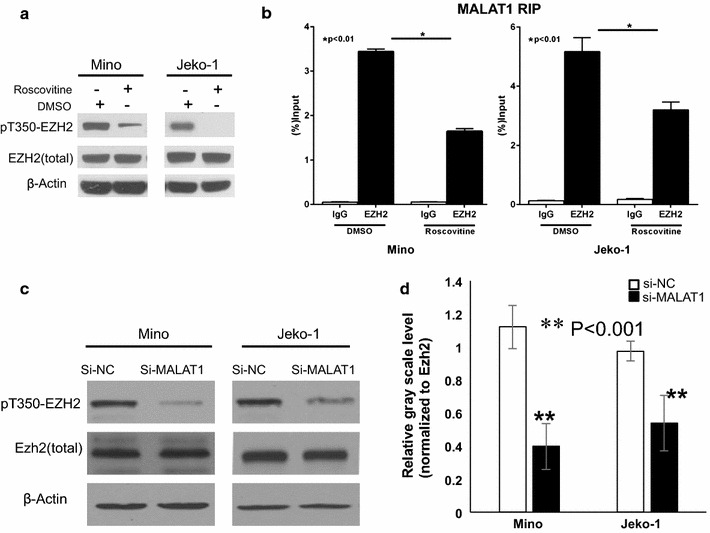
MALAT1 facilitated phosphorylation of EZH2 at T350, which up-regulated MALAT1-binding activity. **a** The effect of CDK inhibitors on EZH2 phosphorylation. Immunoblot analysis of pT350-EZH2, total EZH2 in Mino and Jeko-1 cells following 12 h treatment with roscovitine (25 μM). **b**, **c** Results from qRT-PCR of MALAT1 bound to EZH2 in Mino (**b**) and Jeko-1 (**c**) cells. Signals were normalized by the total amount of MALAT1 mRNA in each case. **d** *Western blot* results revealed the expression of both total EZH2 and pT350-EZH2 significantly decreased in Mino and Jeko-1cells transfected with si-MALAT1. Phosphorylation of EZH2 at Thr 350 was inhibited in MALAT1 knockdown cells, normalized to total EZH2

Some reports have demonstrated that cyclin-dependent kinase (CDK) 1 and CDK2 can induce phosphorylation of EZH2 at threonine 350 (T350) which increased the binding to lncRNAs, such as HOTAIR and XIST, thus further facilitating recruitment of PRC2 to its target genes [25–27]. To investigate whether EZH2 can be phosphorylated at T350 by CDKs in MCL cells, Mino and Jeko-1 cells were treated for 12 h with the CDKs inhibitor (roscovitine, 25 μM). Western blot results showed the phosphorylation of EZH2 at T350 was inhibited with roscovitine (Fig. 6a). To further determine whether the phosphorylation of EZH2 at T350 influence binding to MALAT1, we performed RIP analysis. Results revealed that binding of MALAT1 with EZH2 significantly decreased with absence of T350 phosphorylation due to roscovitine treatment (Mino and Jeko-1; P < 0.01; Fig. 6b). As our data shows that MALAT1 represses the expression of CDKs inhibitors, p21 and p27, we sought to determine whether MALAT1 knockdown could change the phosphorylation of EZH2 at T350. As expected, Western Blot analysis demonstrated that the levels of pT350-EZH2 decreased in MALAT1 knockdown MCL cells (Fig. 6c). To determine whether the decrease of pT350–EZH2 was due to change of total EZH2, the band on Western blot was quantified. We found that pT350–EZH2 levels decreased after normalization to total EZH2 in MALAT1 knockdown MCL cells (Fig. 6d).

**Fig. 7:**
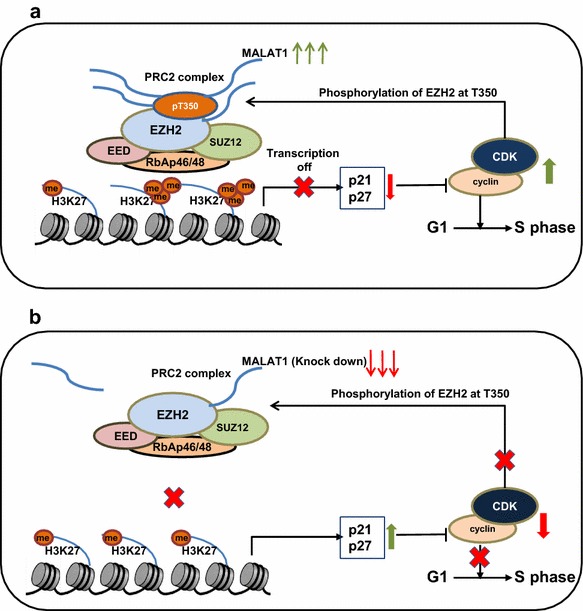
Schematic diagram illustrating signaling of MALAT1 and its downstream effectors in MCL. **a** LncRNA MALAT1 recruits PRC2 to target genes through binding to EZH2. Then, CDKN1A/p21 and CDKN1B/p27 expression is epigenetically repressed by H3K27me3. Then, activated CDK1 and CDK2 promotes phosphorylation of EZH2 at T350, resulting in increase binding to MALAT1. **b** Low expression or knockdown of MALAT1 decrease the recruitment of PRC2 to targeted genes p21 and p27, which inhibit CDKs activities, causing cell cycle arrest and decreased phosphorylation of EZH2 at T350. Finally, EZH2 with low T350 phosphorylation further inhibits its MALAT1-binding activity

Our results suggest that deletion of MALAT1 inhibits recruitment of PRC2 to target genes loci, which further results in re-expressing of CDKs inhibitor, p21 and p27, thereby inducing cell cycle arrest and reduces T350 phosphorylated EZH2, and further resulting in weaker binding to MALAT1 (Fig. 7b).

## Discussion

Previous studies have reported high expression of lncRNA MALAT1 in solid tumors and hematologic malignancies and hinted to its role on transcription complexes [16]. Here we showed that MALAT1 is overexpressed in MCL tissues, which correlates with high MIPI and poor overall survival of MCL patients. Silencing of MALAT1 expression inhibited cell proliferation and increased apoptosis rates of MCL cells. In addition, we observed that down-regulation of MALAT1 increased expression of p21 and p27 through an EZH2-associated mechanism. Moreover, decreased phosphorylation of EZH2 at T350 attenuated its binding to MALAT1. Collectively, our results implicate MALAT1 as a mechanistic and prognostic marker for MCL.

MALAT1 has been found to be up-regulated in several types of solid tumors [16]. Our study investigated MCL, an aggressive type of non Hodgkins lymphoma, and found that the expression of MALAT1 was significantly higher in MCL compared to normal B-cells (P < 0.01). We additionally identified that the increased expression of MALAT1 was associated with high-risk group (by MIPI) and lower overall survival after current chemotherapy in patients with MCL. We probe for the role of MALAT1 using knockdown approach. After siRNA-mediated knockdown of MALAT1, cell proliferation was decreased and the percentage of apoptotic cells was significantly increased in MCL cells (P < 0.01). MALAT1 in another hematological cancer, multiple myeloma (MM) has been shown to also target latent transforming growth factor beta-binding protein 3 (LTB3) and suggest that MALAT1 may have widespread effects across different transcription complexes [28–30]. Mechanistically, MALAT1 recruited the transcription factor Sp1 to the promoter of LTBP3, thereby resulting in elevated level of LTBP3 transcription and TGF-β1 secretion, which plays a role in the suppression of bone formation in MM bone lesions [29]. MALAT1 was also found associated with molecular pathways involving cell cycle regulation, p53-mediated DNA damage response, and mRNA maturation processes in MM using microarray analysis [30].

Our immunoprecipitation experiments revealed notably high affinity of binding between MALAT1 and EZH2, rather than SUZ12 in MCL. Recent studies reported that several lncRNAs (such as HOTAIR, Xist, and H19) act to repress genes by binding PRC2 [31–34]. Further studies of over 3300 lncRNAs identified that about 20% of these lncRNAs are binding partners for PRC2 [35]. Notably, binding of lncRNA to PRC2 is size dependent, with higher affinity belonging to longer RNAs [36]. In line with this, lncRNA MALAT1, with a large size (∼8000 nt), has been found to interact with PRC2 through EZH2 or SUZ12. Specifically, EZH2 mediates the role of the MALAT1–PRC2 partnership in the epithelial–mesenchymal transition in renal cell carcinoma [22], whereas SUZ12 promotes the role of the MALAT1-PRC2 binding in tumor metastasis in bladder cancer [21].

Our results showed that the EZH2 mRNA expression was significantly higher in primary MCL tissues compared to normal B-cells (P < 0.01). Patients with a higher level of EZH2 expression had lower overall survival as compared to those with low EZH2 expression. Our data showed that there was a significant positive correlation between MALAT1 and EZH2 mRNA in MCL. Using Basso’s Lymphoma dataset from Oncomine database, we previously showed that EZH2 mRNA is highly expressed in lymphomas compared to healthy donor’s B-lymphocytes [8]. Independent of tumor proliferation, the expression level of EZH2 was identified as a prognostic factor in MCL, using multivariate survival analysis [37]. Thus, we sought to determine the underlying molecular mechanisms by which MALAT1 functions in concert with EZH2 to regulate downstream effector in MCL.

Through EZH2-mediated mechanisms, PRC2 catalyzes the trimethylation of histone H3 on lysine 27 (i.e. H3K27me3) to repress transcription of specific genes. Upregulation of EZH2 leads to silencing of the genes that are involved in the progression and metastasis of solid tumors [38, 39], and malignant hematopoiesis and lymphoproliferative disorders [40]. Our results revealed that after knockdown of MALAT1, the levels of EZH2 and H3K27me3 expression were both decreased, whereas that of p21 and p27 were increased at both mRNA and protein levels, resulting in cell cycle arrest at G1/S transition. p21 and p27 are both CDKs suppressors involved in regulating cell cycle progression, and have also been considered as candidates for tumor-suppressor genes. Reduced expression of p21 or p27 has shown to be correlated with increased malignancy, high Ki-67 index and poor prognosis in MCL patients [37, 41, 42]. Upregulation of EZH2 was related with cell proliferation in the development of B lymphocyte, and tumor suppressor genes, CDKN1A/p21 and CDKN1B/p27, were histone-3 lysine27-trimethylated and repressed in those proliferating GC-B-cells [24]. And siRNA-mediated knockdown of EZH2 increased the mRNA expression of CDKN1A/p21 and CDKN1B/p27, leading to cell cycle arrest at G1/S transition in DLBCL and MCL cell lines [24, 43]. Consistently, in several solid tumors (such as prostate cancer, ovarian cancer and lung cancer), CDKN1A/p21 and CDKN1B/p27 were identified as EZH2 targets by ChIP-qPCR analysis, and inhibited due to elevated level of EZH2 [44–47].

Intriguingly, some reports have identified an important role for MALAT1 in regulating p21 and p27, potentially mediated by p53 [48, 49]. However, no p53 knockdown experiments were carried out to directly show that whether p53 is the only mediator of p21 regulation by MALAT1 [48]. Indeed, another report found no correlation between the expression of p21 and p53 in MCL patients, suggesting the possibility of p53-independent mechanisms underlying MALAT1-mediated regulation of p21 [50]. A recent study demonstrated that p21 regulation is determined by the methylation and acetylation status of histone H3 on the p21 (WAF-1) promoter in lymphoma [51]. These previous observations lead to our hypothesis that p21 and p27 are regulated by MALAT1 through an EZH2-driven H3K27me3 mechanism in MCL.

In the present study, our ChIP analysis demonstrated that EZH2 occupancy and H3K27me3 level at the promoter domains of p21 and p27 were both significantly decreased in MALAT1-deficient cells compared with control cells (P < 0.01), suggesting that overexpressed MALAT1 down-regulates p21 and p27, a process possibly mediated by EZH2-driven H3K27me3 in MCL. Consistent with our results, EZH2 depletion by either DZNep or siRNA induced the expression of p21 and p27 in MCL cell lines [43].

It has been established that CDK1 and CDK2 phosphorylate EZH2 at T350, which is important for its binding to lncRNAs, HOTAIR and XIST, and recruitment of the PRC2 complex to the EZH2 target genes [25–27]. Our results suggested that the phosphorylation of EZH2 at T350 affected its binding to lncRNA MALAT1. This is consistent with a previous study showing that lncRNA MALAT1 preferentially binds to EZH2 in two regions (amino acids 1–173 and 336–554), including the phosphorylation site T350 [52]. In the present study, we initially observed that the phosphorylation of EZH2 at T350 was consistently reduced after treatment with CDKs inhibitor roscovitine. To confirm consequence of reduced phosphorylation of EZH2 at T350, our RIP results show decreased EZH2–MALAT1 binding.

Our results showed that the phosphorylation of EZH2 at T350 was inhibited in MALAT1-deficient MCL cells. This could be a result of elevated expression of CDKs inhibitor p21 and p27. To collectively explain these observations, we propose a hypothesis of positive feedback loop. Specifically, up-regulated expression of MALAT1 leads to the recruitment of EZH2 to target gene loci, thus enhancing EZH2-mediated H3K27me3 and suppressing the expressions of p21 and p27 (Fig. 7b). This leads to the activation of CDK1 and CDK2 that promote the phosphorylation of EZH2 at T350, which further increases the binding of EZH2 with MALAT1. This increased binding in turn enhances EZH2-mediated H3K27me3 and gene repression (Fig. 7b). Given that CDK1 and CDK2 are highly activated at the S and G2 phase, we envision a model that CDK-induced phosphorylation of EZH2 would probably become decreased at the other phase of the cell cycle in normal cells, which might facilitate the expression of EZH2 target genes and thereby promote cell differentiation. In MCL cells, CDKs are constantly activated due to the repression of p21 and p27 genes by overexpressed lncRNA MALAT1, thereby activating EZH2 to induce uncontrolled cell proliferation. We showed that deletion of MALAT1 with siRNA interferes with this postulated positive feedback loop, resulting in cell cycle arrest.

This study shows that overexpression of the lncRNA MALAT1 provides some oncogenic properties, and may be a prognostic factor or therapeutic target in MCL. MALAT1 expression is significantly higher in MCL tissues than normal tissues (P < 0.01). This may be associated with the key translocation of MCL t (11;14) (q13;q32), the breakpoint of which is adjacent to the MALAT1 gene loci 11q13. Further experiments are required to delineate this hypothesis. Our study also needs to be interpreted with cautions due to the lack of in vivo experiments. Future experiments with appropriate animal models may be helpful to clearly understand the underlying molecular mechanism in MCL progression. Small interfering RNA is a good choice for deleting lncRNAs, which locate in the cytoplasm. But for suppressing nuclear lnRNAs, such as MALAT1 and NEAT1, it is more effective to use antisense oligonucleotides (ASOs) [53]. Preclinical studies have shown the therapeutic efficacy of ASOs targeting MALAT1 in the mouse MMTV–PyMT breast cancer model. Systemic knockdown of MALAT1 through subcutaneous injection of ASOs inhibits tumor proliferation and metastasis, and induces differentiation [54]. Moreover, RNA depletion is not the only way to inhibit lncRNA function. Using steric blocking oligonucleotides, lncRNAs may be blocked to interact with their binding partners, such as protein, DNA and miRNA [55]. Future technical innovations will offer more effective lncRNA-targeting therapeutics.

## Conclusions

We first report that MALAT1 is highly expressed in MCL cells and correlated with clinic characters including patient outcome. After MALAT1 knockdown, cell proliferation was inhibited and apoptosis rate was increased in MCL cell lines, suggesting that MALAT1 could function as a potential oncogene in MCL. Our results clearly demonstrate that knockdown of MALAT1 reduced EZH2 level and recruitment to the target gene (p21 and p27), resulting in cell cycle arrest at G1/S transition. We subsequently show that MALAT1-induced EZH2 recruitment is self-enhanced through EZH2 phosphorylation at T350 in MCL. Above all, MALAT1 may be used as a prognostic factor or therapeutic target in MCL.

## Additional files

**Additional file 1: Table S1.** Association of MALAT1 expression with clinical parameters of MCL patients.

**Additional file 2: Figure S1.** Basal expression level of MALAT1 in MCL cell lines. Relative MALAT1 expression in MCL derived cell lines Z-138, Mino, REC-1, Jeko-1, JVM2 and Granta-519 were measured by qRT-PCR and normalized to gene expression levels of GAPDH. The expression of MALAT1 was significantly higher in Mino and Jeko-1 cells, which were used in additional experiments.

**Additional file 3: Figure S2.** Knockdown of MALAT1 inhibited proliferation of MCL. **a** Knockdown of MALAT1 with si-MALAT1(No.235 and 236) and nontarget control analyzed by qRT-PCR. **b** Cell viability assay using MTT on MCL cells transfected with si-MALAT1 No. 236 or si-NC.

**Additional file 4: Figure S3.** Representative images of colony formation of Mino cells. **a** The number of colonies in MALAT1 knockdown Mino cells were significantly reduced. **b** Photographs of colonies in colony formation assay. The size of individual colony was significantly reduced in MALAT1 knockdown Mino cells.

**Additional file 5: Figure S4.** MALAT1 knockdown enhances MCL cell apoptosis in vitro. In flow cytometric analysis of annexin V/PI staining, the percentage of either early or late apoptotic cells increases in MALAT1 knock down cells.

**Additional file 6: Figure S5.** Knockdown of MALAT1 enhanced apoptosis. Apoptosis in MCL cells transfected with si-MALAT1 No. 236 or si-NC was detected by flow cytometry after annexin V/PI staining.

**Additional file 7: Figure S6.** Knockdown of MALAT1 and cell cycle profile. Cell cycle analysis of control (si-NC) and MALAT1 knockdown cells (si-MALAT1) post-mitotic release. **a** Transfected MCL cells (Mino and Jeko-1) were treated with nocodazole for 24 hours for synchronization and examined for G1/S progression 18 hours after released. **b** Cell cycle analysis of control and MALAT1 knockdown cells by flow cytometry.

**Additional file 8: Figure S7.** Cell cycle analysis by flow cytometry. Ratio of cell percentage in S phase to G1 phase significantly decreased in MALAT1 knockdown MCL cells (Mino and Jeko-1). Data are representative of three independent experiments and represent the mean ± SD.

**Additional file 9: Table S2.** Association of EZH2 expression with clinical parameters of MCL patients.

**Additional file 10: Figure S8.** Effect of MALAT1 knocking down on the expression of EZH2 and H3k27me3, and cell cycle regulators p21 and p27. The expressions of EZH2 and H3k27me3 were moderately suppressed, while p21 and p27 were increased in MALAT1 knock down cells (Mino and Jeko-1) as analyzed by Western blot.

## Abbreviations

lncRNAs: long non-coding RNAs
MALAT1: metastasis-associated lung adenocarcinoma transcript 1
MCL: mantle cell lymphoma
EZH2: enhancer of zeste homolog 2
MIPI: MCL international prognostic index
qRT-PCR: quantitative real time polymerase chain reaction
NHL: non-Hodgkin’s lymphoma
DLBCL: diffuse large B cell lymphoma
H3K27me3: tri-methylation of histone H3 on lysine 27
PRC2: polycomb repressive complex2
RIP: RNA Immunoprecipitation
GCB: germinal center B
ChIP: chromatin immunoprecipitation
MM: multiple myeloma
ASOs: antisense oligonucleotides

## References

[1] Zhou Y, Wang H, Fang W, Romaguer JE, Zhang Y, Delasalle KB, Kwak L, Yi Q, Du XL, Wang M (2008). Incidence trends of mantle cell lymphoma in the United States between 1992 and 2004. Cancer.

[2] Cheah CY, Seymour JF, Wang ML (2016). Mantle cell lymphoma. J Clin Oncol.

[3] Djebali S, Davis CA, Merkel A, Dobin A, Lassmann T, Mortazavi A, Tanzer A, Lagarde J, Lin W, Schlesinger F (2012). Landscape of transcription in human cells. Nature.

[4] Ponting CP, Oliver PL, Reik W (2009). Evolution and functions of long noncoding RNAs. Cell.

[5] Zhang H, Chen Z, Wang X, Huang Z, He Z, Chen Y (2013). Long non-coding RNA: a new player in cancer. J Hematol Oncol..

[6] Guttman M, Amit I, Garber M, French C, Lin MF, Feldser D, Huarte M, Zuk O, Carey BW, Cassady JP (2009). Chromatin signature reveals over a thousand highly conserved large non-coding RNAs in mammals. Nature.

[7] Schmitt AM, Chang HY (2016). Long noncoding RNAs in cancer pathways. Cancer Cell.

[8] Sehgal L, Mathur R, Braun FK, Wise JF, Berkova Z, Neelapu S, Kwak LW, Samaniego F (2014). FAS-antisense 1 lncRNA and production of soluble versus membrane Fas in B-cell lymphoma. Leukemia.

[9] Peng W, Fan H, Wu G, Wu J, Feng J (2016). Upregulation of long noncoding RNA PEG10 associates with poor prognosis in diffuse large B cell lymphoma with facilitating tumorigenicity. Clin Exp Med..

[10] Peng W, Feng J (2016). Long noncoding RNA LUNAR1 associates with cell proliferation and predicts a poor prognosis in diffuse large B-cell lymphoma. Biomed Pharmacother.

[11] Peng W, Wu J, Feng J (2016). Long noncoding RNA HULC predicts poor clinical outcome and represents pro-oncogenic activity in diffuse large B-cell lymphoma. Biomed Pharmacother.

[12] Verma A, Jiang Y, Du W, Fairchild L, Melnick A, Elemento O (2015). Transcriptome sequencing reveals thousands of novel long non-coding RNAs in B cell lymphoma. Genome Med..

[13] Pan Y, Li H, Guo Y, Luo Y, Li H, Xu Y, Deng J, Sun B (2016). A pilot study of long noncoding RNA expression profiling by microarray in follicular lymphoma. Gene.

[14] Ma XY, Wang JH, Wang JL, Ma CX, Wang XC, Liu FS (2015). Malat1 as an evolutionarily conserved lncRNA, plays a positive role in regulating proliferation and maintaining undifferentiated status of early-stage hematopoietic cells. BMC Genom.

[15] Kato L, Begum NA, Burroughs AM, Doi T, Kawai J, Daub CO, Kawaguchi T (2012). Nonimmunoglobulin target loci of activation-induced cytidine deaminase (AID) share unique features with immunoglobulin genes. Proc Natl Acad Sci USA.

[16] Yoshimoto R, Mayeda A, Yoshida M, Nakagawa S (2016). MALAT1 long non-coding RNA in cancer. Biochim Biophys Acta.

[17] George SK, Vishwamitra D, Manshouri R, Shi P, Amin HM (2014). The ALK inhibitor ASP3026 eradicates NPM–ALK(+) T-cell anaplastic large-cell lymphoma in vitro and in a systemic xenograft lymphoma model. Oncotarget..

[18] Wise JF, Berkova Z, Mathur R, Zhu H, Braun FK, Tao RH, Sabichi AL, Ao X, Maeng H, Samaniego F (2013). Nucleolin inhibits Fas ligand binding and suppresses Fas-mediated apoptosis in vivo via a surface nucleolin-Fas complex. Blood.

[19] Hoster E, Dreyling M, Klapper W, Gisselbrecht C, van Hoof A, Kluin-Nelemans HC, Pfreundschuh M, Reiser M, Metzner B, Einsele H (2008). A new prognostic index (MIPI) for patients with advanced-stage mantle cell lymphoma. Blood.

[20] Margueron R, Reinberg D (2011). The Polycomb complex PRC2 and its mark in life. Nature.

[21] Fan Y, Shen B, Tan M, Mu X, Qin Y, Zhang F, Liu Y (2014). TGF-beta-induced upregulation of malat1 promotes bladder cancer metastasis by associating with suz12. Clin Cancer Res.

[22] Hirata H, Hinoda Y, Shahryari V, Deng G, Nakajima K, Tabatabai ZL, Ishii N, Dahiya R (2015). Long Noncoding RNA MALAT1 Promotes Aggressive Renal Cell Carcinoma through Ezh2 and Interacts with miR-205. Cancer Res.

[23] Zhang E, He X, Yin D, Han L, Qiu M, Xu T, Xia R, Xu L, Yin R, De W (2016). Increased expression of long noncoding RNA TUG1 predicts a poor prognosis of gastric cancer and regulates cell proliferation by epigenetically silencing of p57. Cell Death Dis.

[24] Velichutina I, Shaknovich R, Geng H, Johnson NA, Gascoyne RD, Melnick AM, Elemento O (2010). EZH2-mediated epigenetic silencing in germinal center B cells contributes to proliferation and lymphomagenesis. Blood.

[25] Chen S, Bohrer LR, Rai AN, Pan Y, Gan L, Zhou X, Bagchi A, Simon JA, Huang H (2010). Cyclin-dependent kinases regulate epigenetic gene silencing through phosphorylation of EZH2. Nat Cell Biol.

[26] Kaneko S, Li G, Son J, Xu CF, Margueron R, Neubert TA, Reinberg D (2010). Phosphorylation of the PRC2 component Ezh2 is cell cycle-regulated and up-regulates its binding to ncRNA. Genes Dev.

[27] Zeng X, Chen S, Huang H (2011). Phosphorylation of EZH2 by CDK1 and CDK2: a possible regulatory mechanism of transmission of the H3K27me3 epigenetic mark through cell divisions. Cell Cycle.

[28] Cho SF, Chang YC, Chang CS, Lin SF, Liu YC, Hsiao HH, Chang JG, Liu TC (2014). MALAT1 long non-coding RNA is overexpressed in multiple myeloma and may serve as a marker to predict disease progression. BMC Cancer.

[29] Li B, Chen P, Qu J, Shi L, Zhuang W, Fu J, Li J, Zhang X, Sun Y, Zhuang W (2014). Activation of LTBP3 gene by a long noncoding RNA (lncRNA) MALAT1 transcript in mesenchymal stem cells from multiple myeloma. J Biol Chem.

[30] Ronchetti D, Agnelli L, Taiana E, Galletti S, Manzoni M, Todoerti K, Musto P, Strozzi F, Neri A (2016). Distinct lncRNA transcriptional fingerprints characterize progressive stages of multiple myeloma. Oncotarget..

[31] Kogo R, Shimamura T, Mimori K, Kawahara K, Imoto S, Sudo T, Tanaka F, Shibata K, Suzuki A, Komune S (2011). Long noncoding RNA HOTAIR regulates polycomb-dependent chromatin modification and is associated with poor prognosis in colorectal cancers. Cancer Res.

[32] Luo M, Li Z, Wang W, Zeng Y, Liu Z, Qiu J (2013). Long non-coding RNA H19 increases bladder cancer metastasis by associating with EZH2 and inhibiting E-cadherin expression. Cancer Lett.

[33] Sarma K, Cifuentes-Rojas C, Ergun A, Del Rosario A, Jeon Y, White F, Sadreyev R, Lee JT (2014). ATRX directs binding of PRC2 to Xist RNA and Polycomb targets. Cell.

[34] Tsai MC, Manor O, Wan Y, Mosammaparast N, Wang JK, Lan F, Shi Y, Segal E, Chang HY (2010). Long noncoding RNA as modular scaffold of histone modification complexes. Science.

[35] Khalil AM, Guttman M, Huarte M, Garber M, Raj A, Rivea Morales D, Thomas K, Presser A, Bernstein BE, van Oudenaarden A (2009). Many human large intergenic noncoding RNAs associate with chromatin-modifying complexes and affect gene expression. Proc Natl Acad Sci USA.

[36] Davidovich C, Zheng L, Goodrich KJ, Cech TR (2013). Promiscuous RNA binding by Polycomb repressive complex 2. Nat Struct Mol Biol.

[37] Kienle D, Katzenberger T, Ott G, Saupe D, Benner A, Kohlhammer H, Barth TF, Holler S, Kalla J, Rosenwald A (2007). Quantitative gene expression deregulation in mantle-cell lymphoma: correlation with clinical and biologic factors. J Clin Oncol.

[38] Zingg D, Debbache J, Schaefer SM, Tuncer E, Frommel SC, Cheng P, Arenas-Ramirez N, Haeusel J, Zhang Y, Bonalli M (2015). The epigenetic modifier EZH2 controls melanoma growth and metastasis through silencing of distinct tumour suppressors. Nat Commun..

[39] Kim KH, Roberts CW (2016). Targeting EZH2 in cancer. Nat Med.

[40] Lund K, Adams PD, Copland M (2014). EZH2 in normal and malignant hematopoiesis. Leukemia.

[41] Pinyol M, Hernandez L, Cazorla M, Balbin M, Jares P, Fernandez PL, Montserrat E, Cardesa A, Lopez-Otin C, Campo E (1997). Deletions and loss of expression of p16INK4a and p21Waf1 genes are associated with aggressive variants of mantle cell lymphomas. Blood.

[42] Letestu R, Ugo V, Valensi F, Radford-Weiss I, Nataf J, Levy V, Gribben JG, Troussard X, Ajchenbaum-Cymbalista F (2004). Prognostic impact of p27KIP1 expression in cyclin D1 positive lymphoproliferative disorders. Leukemia.

[43] Fiskus W, Rao R, Balusu R, Ganguly S, Tao J, Sotomayor E, Mudunuru U, Smith JE, Hembruff SL, Atadja P (2012). Superior efficacy of a combined epigenetic therapy against human mantle cell lymphoma cells. Clin Cancer Res.

[44] Ding L, Chen S, Liu P, Pan Y, Zhong J, Regan KM, Wang L, Yu C, Rizzardi A, Cheng L (2014). CBP loss cooperates with PTEN haploinsufficiency to drive prostate cancer: implications for epigenetic therapy. Cancer Res.

[45] Seward S, Semaan A, Qazi AM, Gruzdyn OV, Chamala S, Bryant CC, Kumar S, Cameron D, Sethi S, Ali-Fehmi R (2013). EZH2 blockade by RNA interference inhibits growth of ovarian cancer by facilitating re-expression of p21 (waf1/cip1) and by inhibiting mutant p53. Cancer Lett.

[46] Negishi M, Wongpalee SP, Sarkar S, Park J, Lee KY, Shibata Y, Reon BJ, Abounader R, Suzuki Y, Sugano S, Dutta A (2014). A new lncRNA, APTR, associates with and represses the CDKN1A/p21 promoter by recruiting polycomb proteins. PLoS ONE.

[47] Qiu M, Xu Y, Wang J, Zhang E, Sun M, Zheng Y, Li M, Xia W, Feng D, Yin R, Xu L (2015). A novel lncRNA, LUADT1, promotes lung adenocarcinoma proliferation via the epigenetic suppression of p27. Cell Death Dis.

[48] Tripathi V, Shen Z, Chakraborty A, Giri S, Freier SM, Wu X, Zhang Y, Gorospe M, Prasanth SG, Lal A, Prasanth KV (2013). Long noncoding RNA MALAT1 controls cell cycle progression by regulating the expression of oncogenic transcription factor B-MYB. PLoS Genet.

[49] Wang X, Li M, Wang Z, Han S, Tang X, Ge Y, Zhou L, Zhou C, Yuan Q, Yang M (2015). Silencing of long noncoding RNA MALAT1 by miR-101 and miR-217 inhibits proliferation, migration, and invasion of esophageal squamous cell carcinoma cells. J Biol Chem.

[50] Chiarle R, Budel LM, Skolnik J, Frizzera G, Chilosi M, Corato A, Pizzolo G, Magidson J, Montagnoli A, Pagano M (2000). Increased proteasome degradation of cyclin-dependent kinase inhibitor p27 is associated with a decreased overall survival in mantle cell lymphoma. Blood.

[51] Escoubet-Lozach L, Lin IL, Jensen-Pergakes K, Brady HA, Gandhi AK, Schafer PH, Muller GW, Worland PJ, Chan KW, Verhelle D (2009). Pomalidomide and lenalidomide induce p21 WAF-1 expression in both lymphoma and multiple myeloma through a LSD1-mediated epigenetic mechanism. Cancer Res.

[52] Wang D, Ding L, Wang L, Zhao Y, Sun Z, Karnes RJ, Zhang J, Huang H (2015). LncRNA MALAT1 enhances oncogenic activities of EZH2 in castration-resistant prostate cancer. Oncotarget..

[53] Lennox KA, Behlke MA (2016). Cellular localization of long non-coding RNAs affects silencing by RNAi more than by antisense oligonucleotides. Nucleic Acids Res.

[54] Arun G, Diermeier S, Akerman M, Chang KC, Wilkinson JE, Hearn S, Kim Y, MacLeod AR, Krainer AR, Norton L (2016). Differentiation of mammary tumors and reduction in metastasis upon Malat1 lncRNA loss. Genes Dev.

[55] Kole R, Krainer AR, Altman S (2012). RNA therapeutics: beyond RNA interference and antisense oligonucleotides. Nat Rev Drug Discov..

