# Supplementary material for: LncRNA MALAT1 promotes development of mantle cell lymphoma by associating with EZH2
Source: J Transl Med. 2016 Dec 20;14:346. doi: 10.1186/s12967-016-1100-9 (PMC5175387; doi:10.1186/s12967-016-1100-9)

Additional file **2**: Figure S1

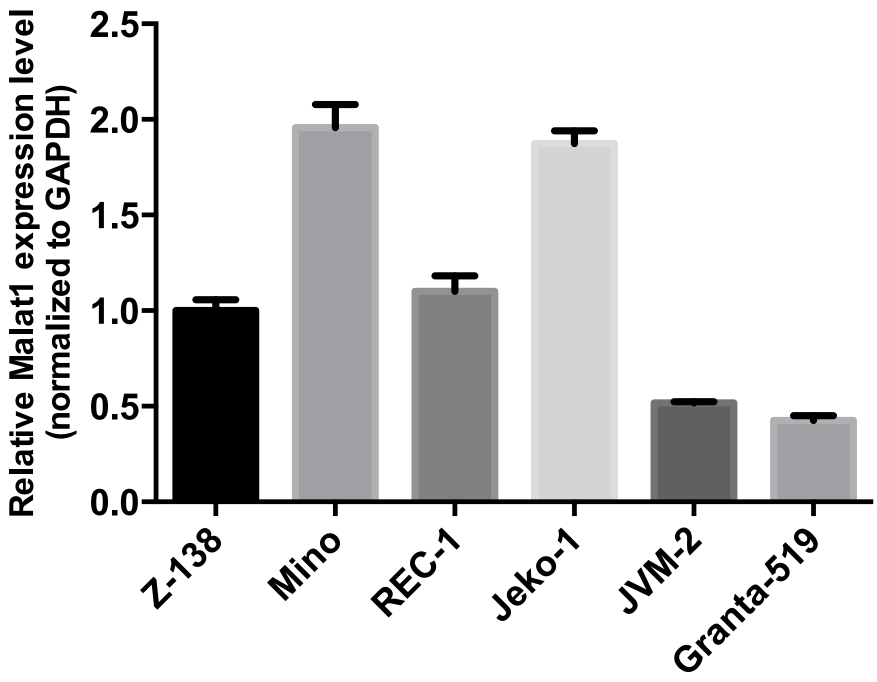

Additional file **3**: Figure S2

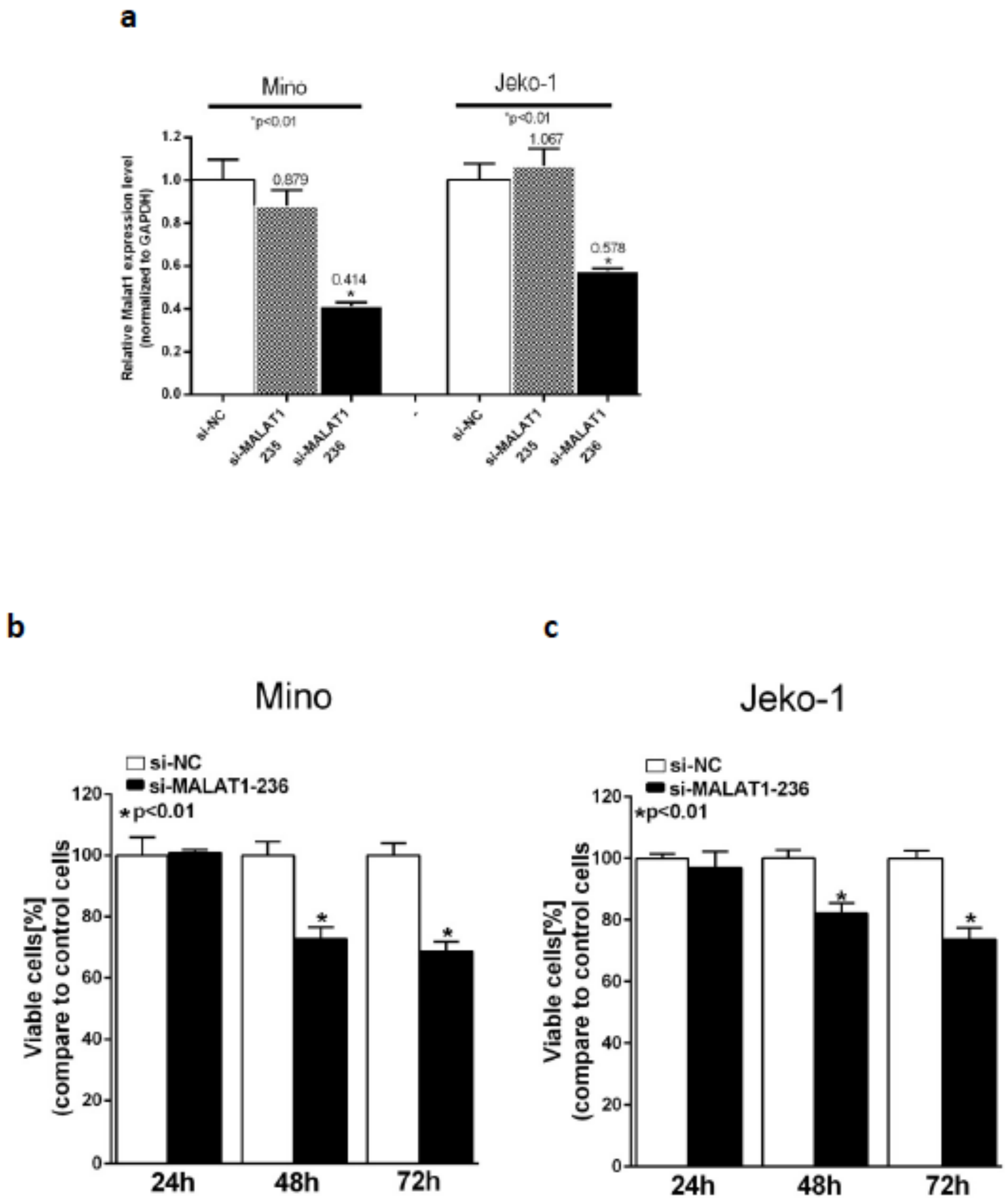

Additional file **4**: Figure S3

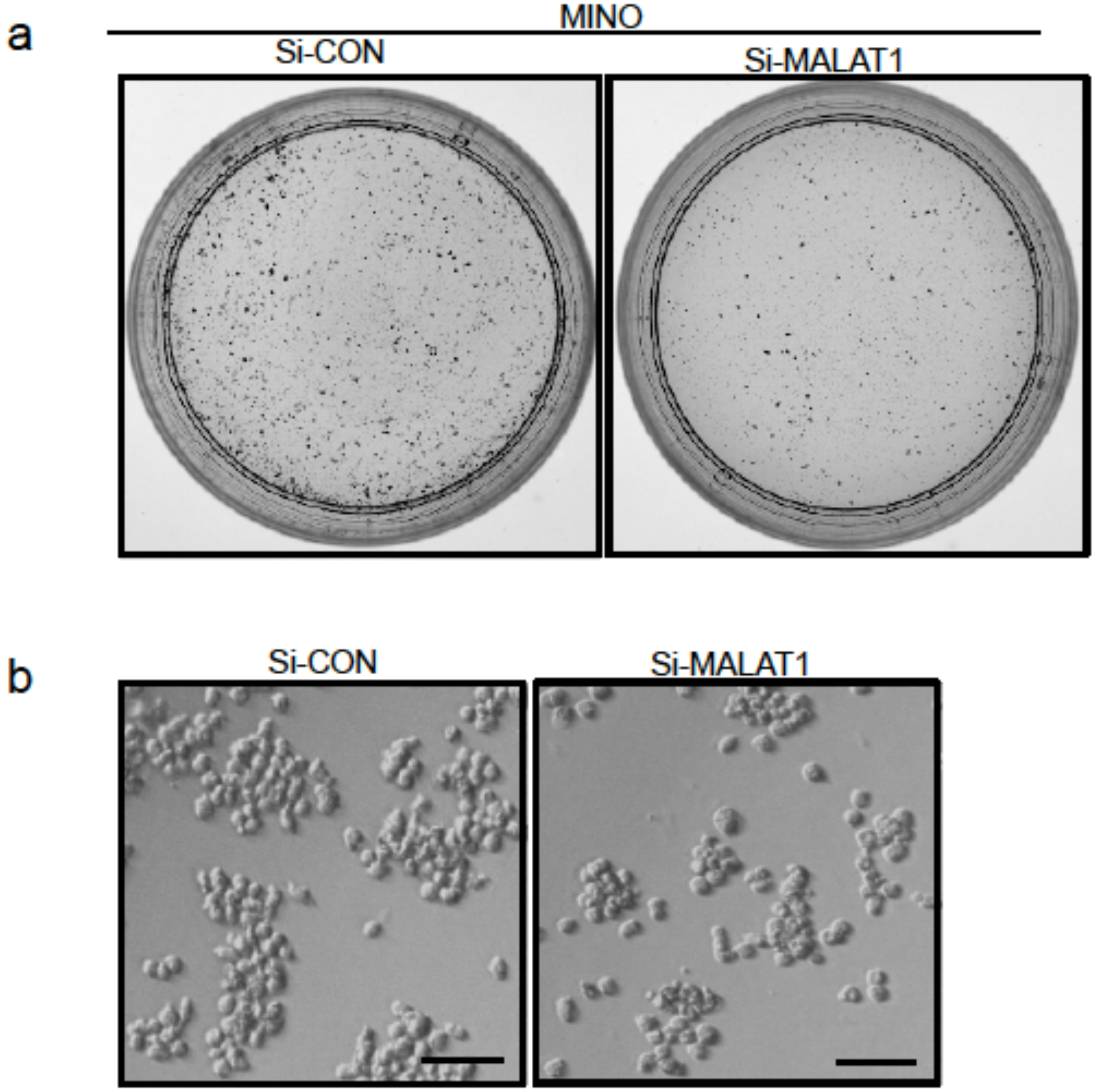

Additional file **5**: Figure S4

%	Mino si-NC	Mino si-Malat1	Jeko-1 si-NC	Jeko-1 si-Malat1
Early Apoptosis	2.35	4.49	0.72	1.32
Late Apoptosis	7.89	13.99	6.36	15.98
Living Cells	88.85	78.72	92.66	82.31
Necrosis	0.90	2.80	0.29	0.38

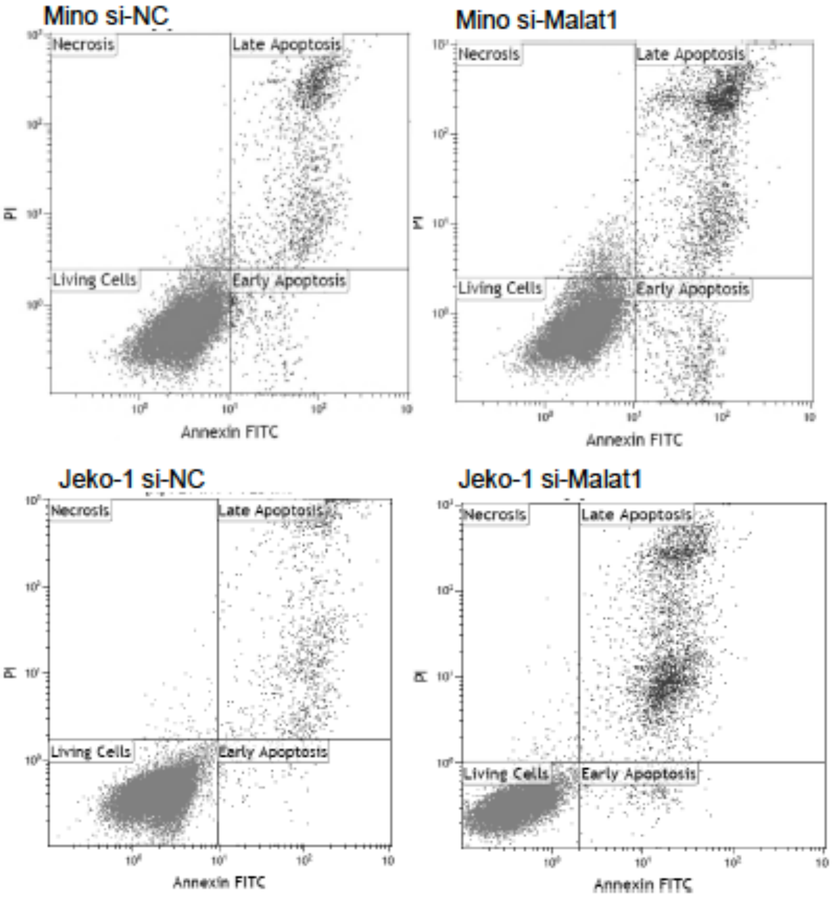

Additional file **6**: Figure S5

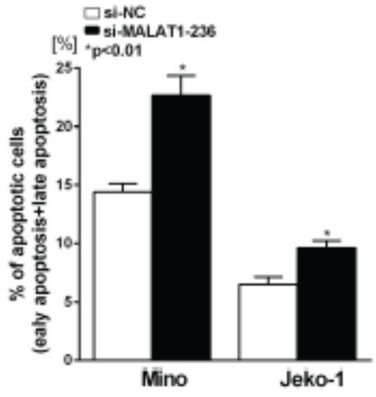

%	Mino si-NC	Mino si-Malat1-236	Jeko-1 si-NC	Jeko-1 si-Malat1
Early Apoptosis	7.55	9.89	2.82	3.52
Late Apoptosis	7.15	13.13	3.56	5.95
Living Cells	84.72	74.65	93.25	89.36
Necrosis	0.58	2.33	0.37	1.18

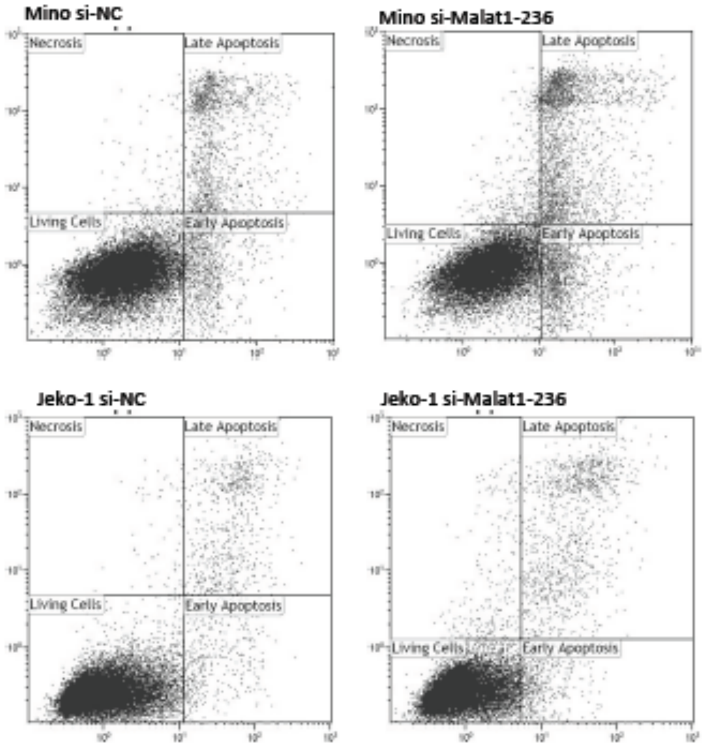

Additional file **7**: Figure S6

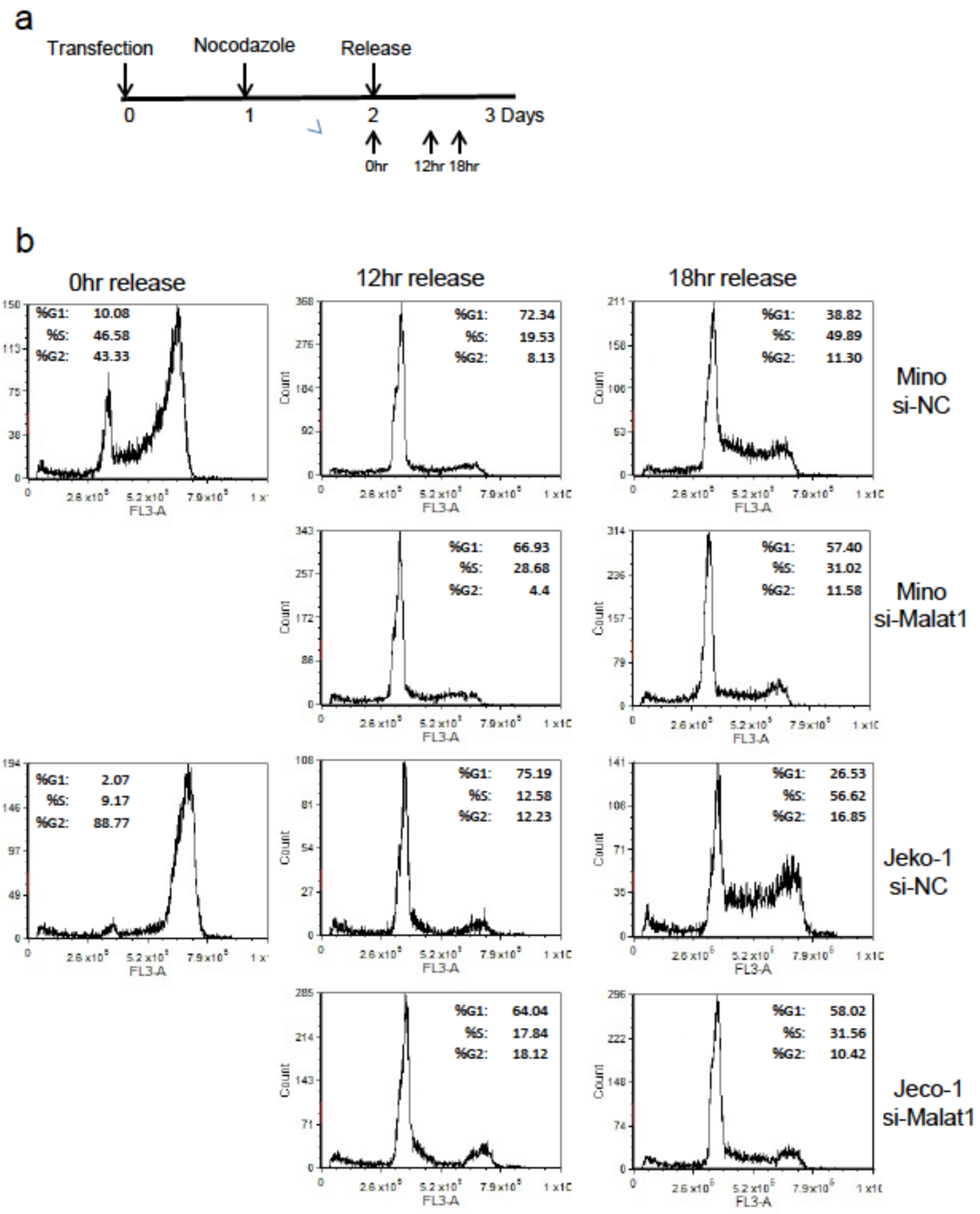

Additional file **8**: Figure S7

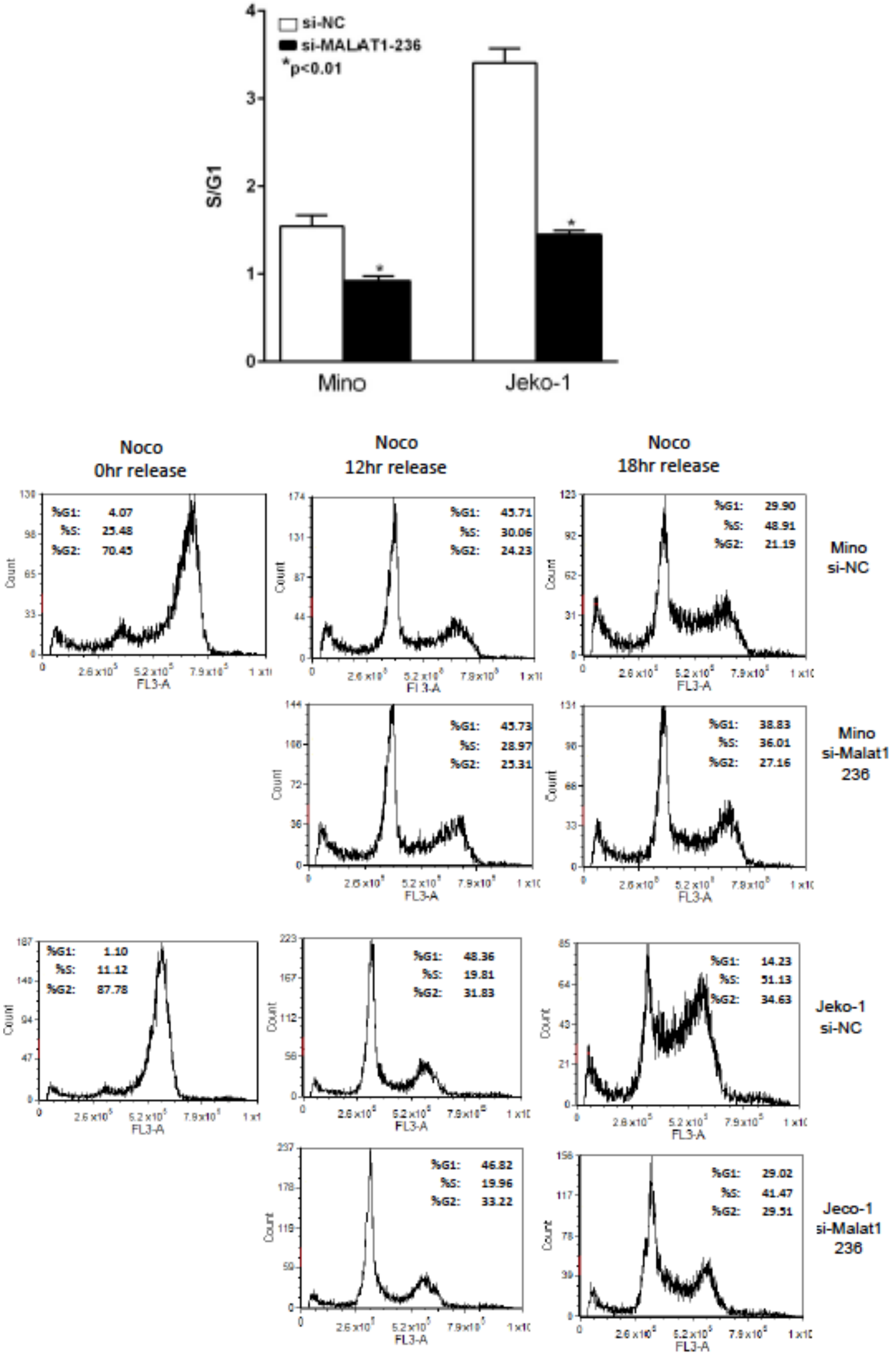

Additional file **10**: Figure S8

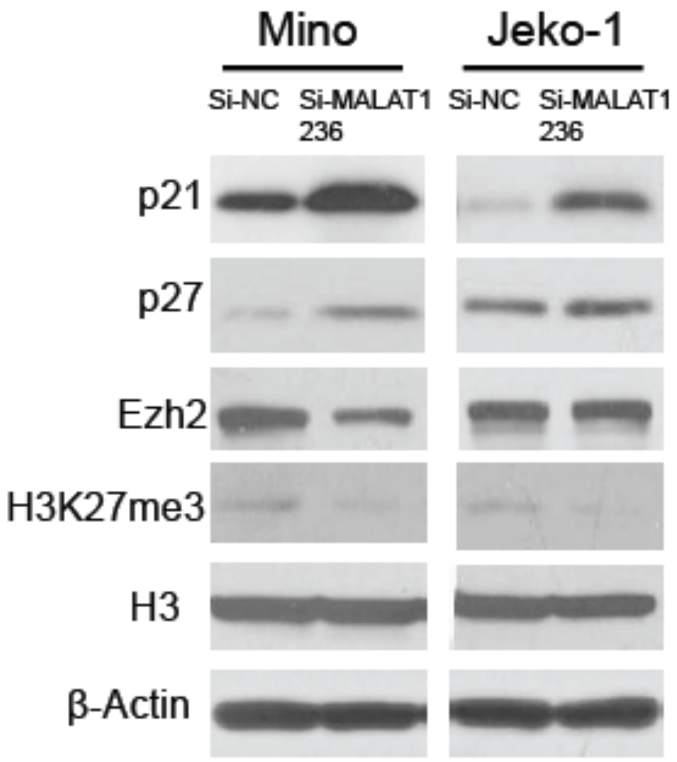

Supplement: Supplementary file 2 — Additional file 2: Figure S1. Basal expression level of MALAT1 in MCL cell lines. Relative MALAT1 expression in MCL derived cell lines Z-138, Mino, REC-1, Jeko-1, JVM2 and Granta-519 were measured by qRT-PCR and normalized to gene expression levels of GAPDH. The expression of MALAT1 was significantly higher in Mino and Jeko-1 cells, which were used in additional experiments. [file 12967_2016_1100_MOESM2_ESM.pdf]
