# Supplementary figures and images for: LncRNA MALAT1 promotes development of mantle cell lymphoma by associating with EZH2
Source: J Transl Med. 2016 Dec 20;14:346. doi: 10.1186/s12967-016-1100-9 (PMC5175387; doi:10.1186/s12967-016-1100-9)

Additional file 3: Figure S2

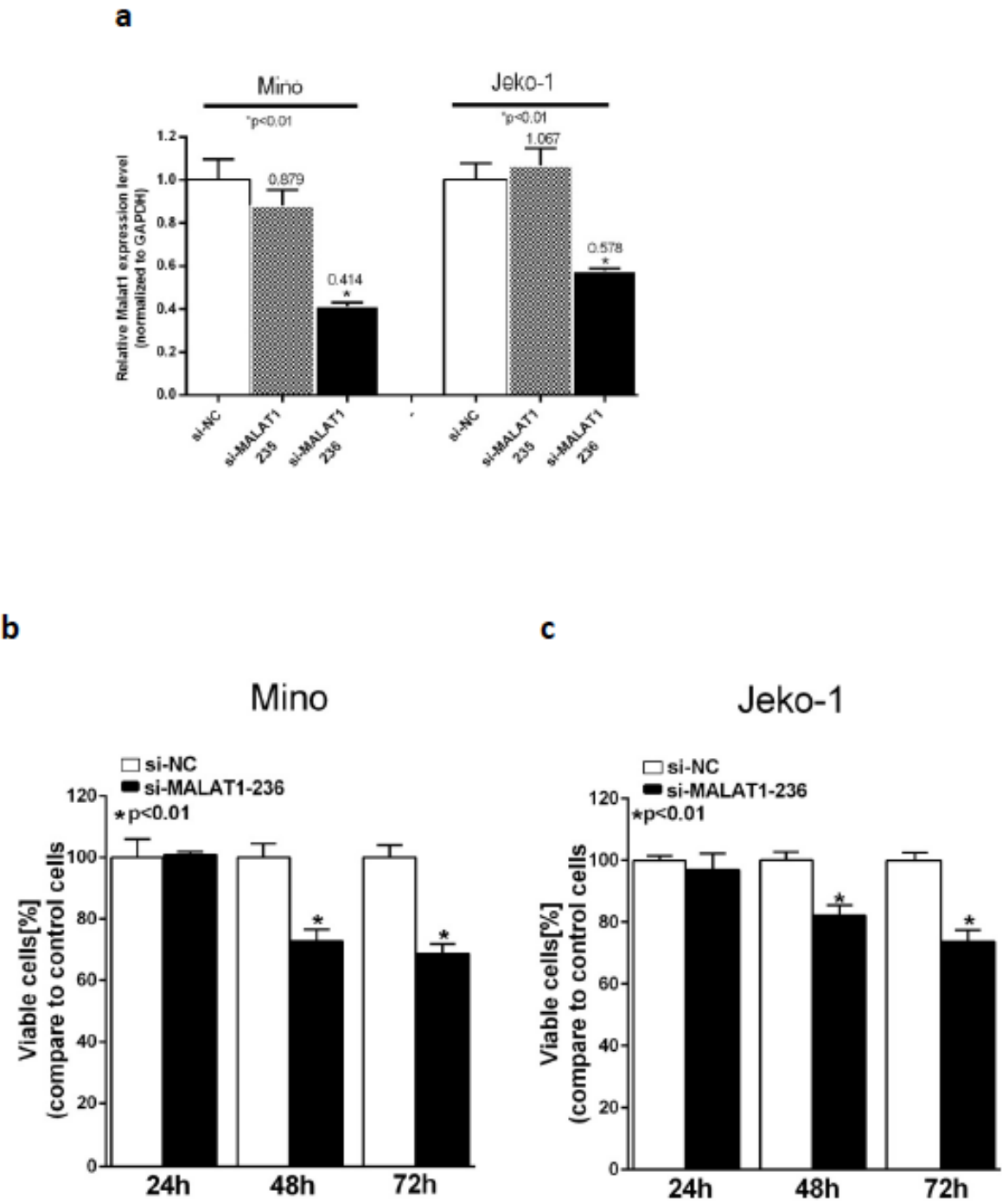

Supplement: Supplementary file 3 — Additional file 3: Figure S2. Knockdown of MALAT1 inhibited proliferation of MCL. a Knockdown of MALAT1 with si-MALAT1(No.235 and 236) and nontarget control analyzed by qRT-PCR. b Cell viability assay using MTT on MCL cells transfected with si-MALAT1 No. 236 or si-NC. [file 12967_2016_1100_MOESM3_ESM.pdf]

Additional file 4: Figure S3

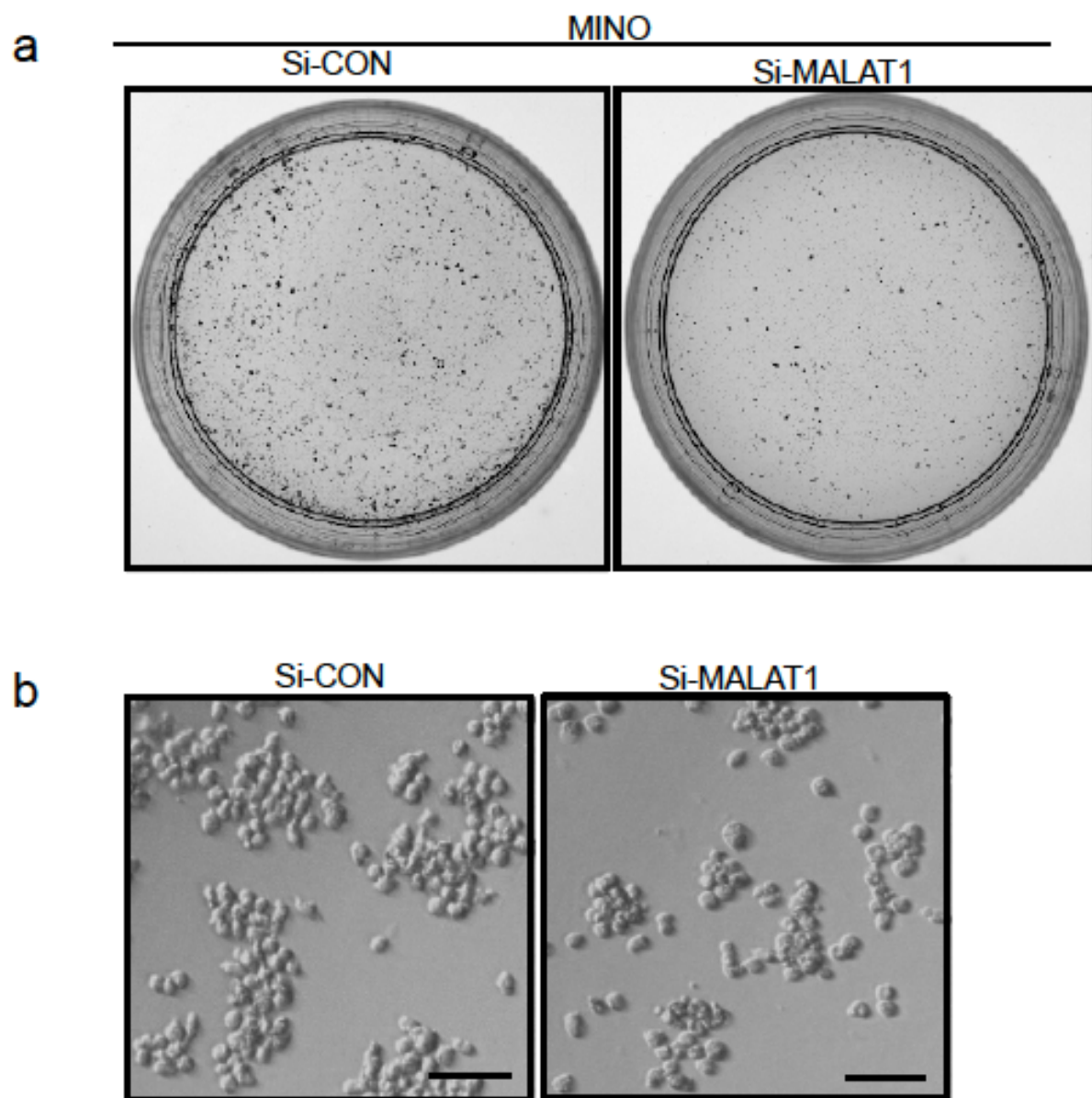

Supplement: Supplementary file 4 — Additional file 4: Figure S3. Representative images of colony formation of Mino cells. a The number of colonies in MALAT1 knockdown Mino cells were significantly reduced. b Photographs of colonies in colony formation assay. The size of individual colony was significantly reduced in MALAT1 knockdown Mino cells. [file 12967_2016_1100_MOESM4_ESM.pdf]

Additional file 6: Figure S5

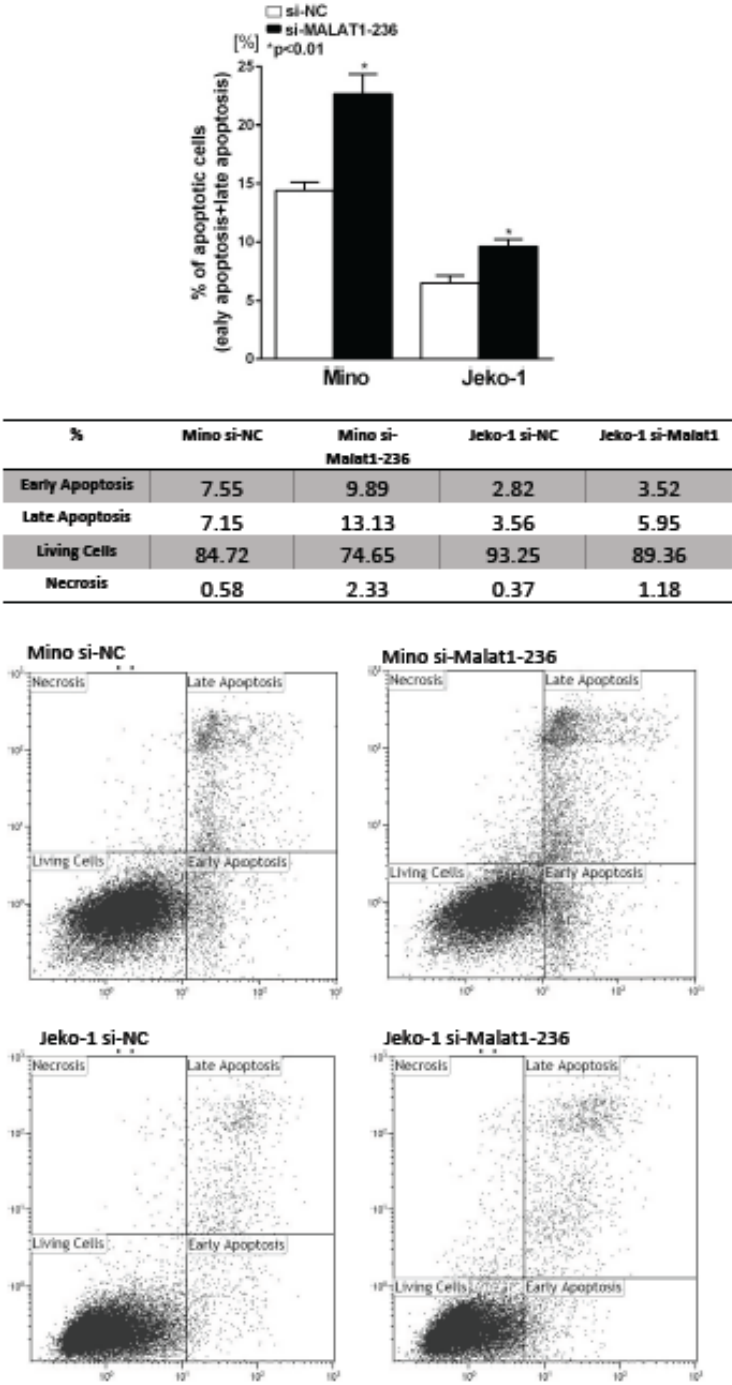

Supplement: Supplementary file 6 — Additional file 6: Figure S5. Knockdown of MALAT1 enhanced apoptosis. Apoptosis in MCL cells transfected with si-MALAT1 No. 236 or si-NC was detected by flow cytometry after annexin V/PI staining. [file 12967_2016_1100_MOESM6_ESM.pdf]

# Additional file 7: Figure S6

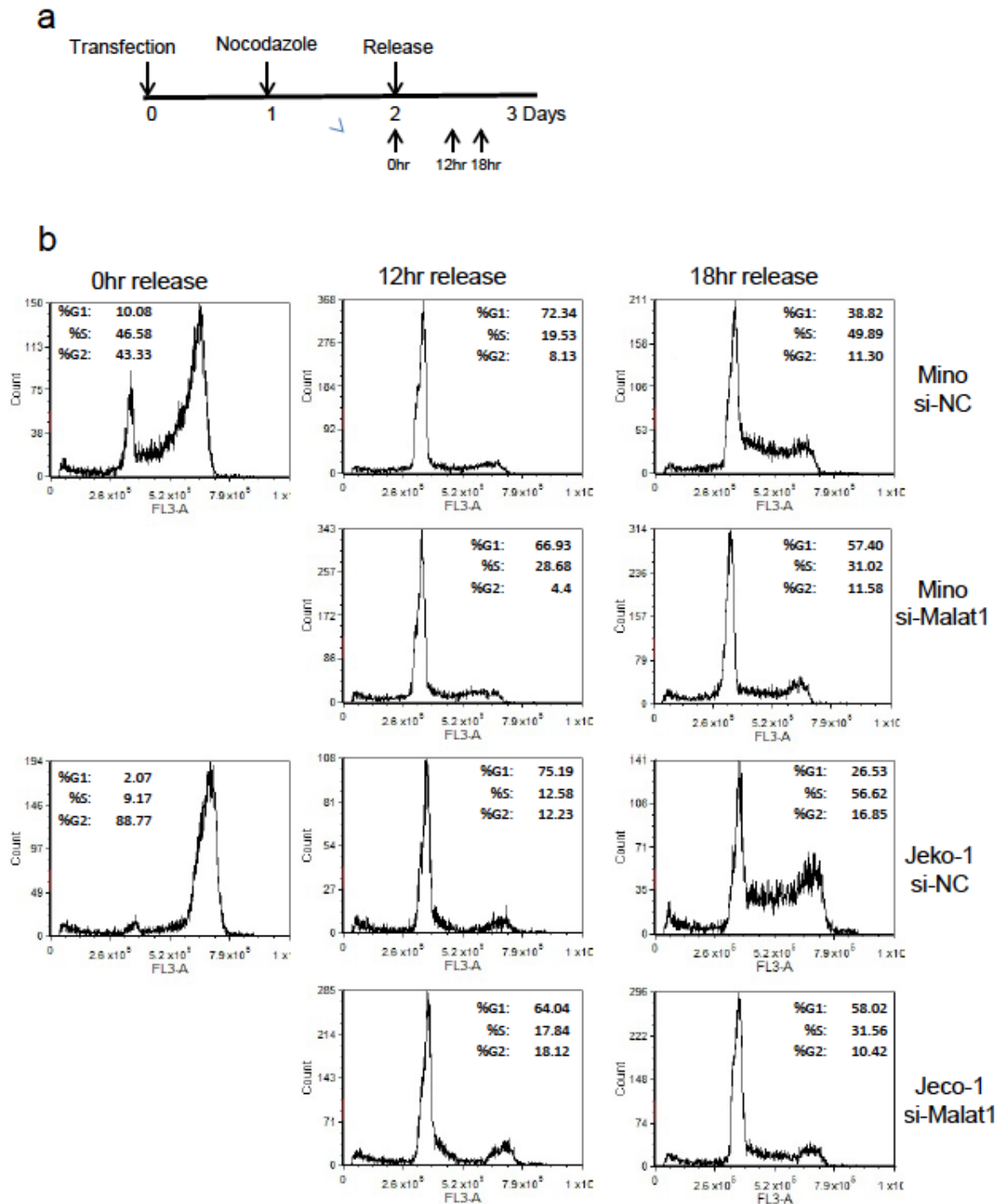

Supplement: Supplementary file 7 — Additional file 7: Figure S6. Knockdown of MALAT1 and cell cycle profile. Cell cycle analysis of control (si-NC) and MALAT1 knockdown cells (si-MALAT1) post-mitotic release. a Transfected MCL cells (Mino and Jeko-1) were treated with nocodazole for 24 hours for synchronization and examined for G1/S progression 18 hours after released. b Cell cycle analysis of control and MALAT1 knockdown cells by flow cytometry. [file 12967_2016_1100_MOESM7_ESM.pdf]

Additional file 8: Figure S7

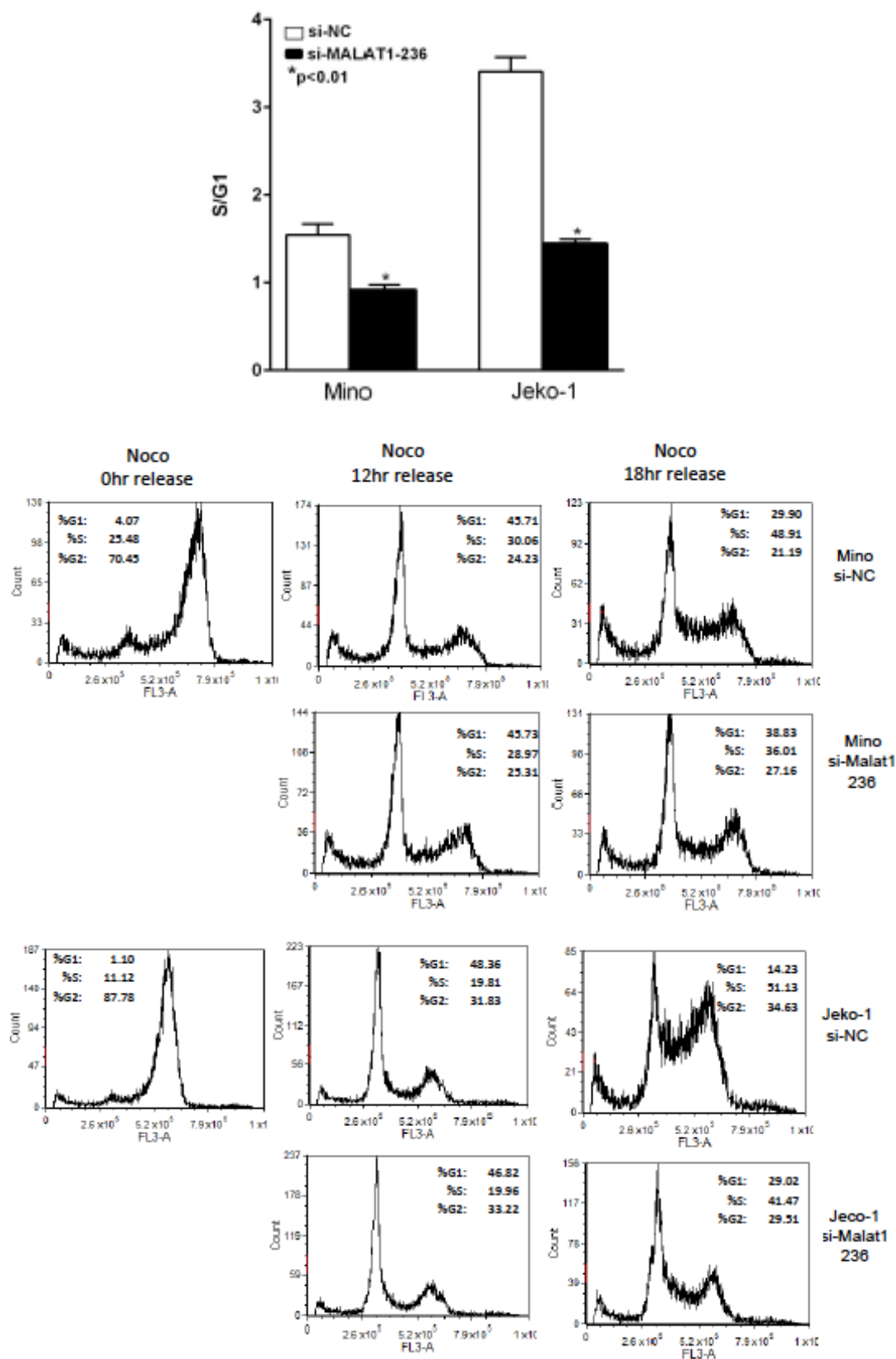

Supplement: Supplementary file 8 — Additional file 8: Figure S7. Cell cycle analysis by flow cytometry. Ratio of cell percentage in S phase to G1 phase significantly decreased in MALAT1 knockdown MCL cells (Mino and Jeko-1). Data are representative of three independent experiments and represent the mean ± SD. [file 12967_2016_1100_MOESM8_ESM.pdf]

Additional file **10**: Figure S8

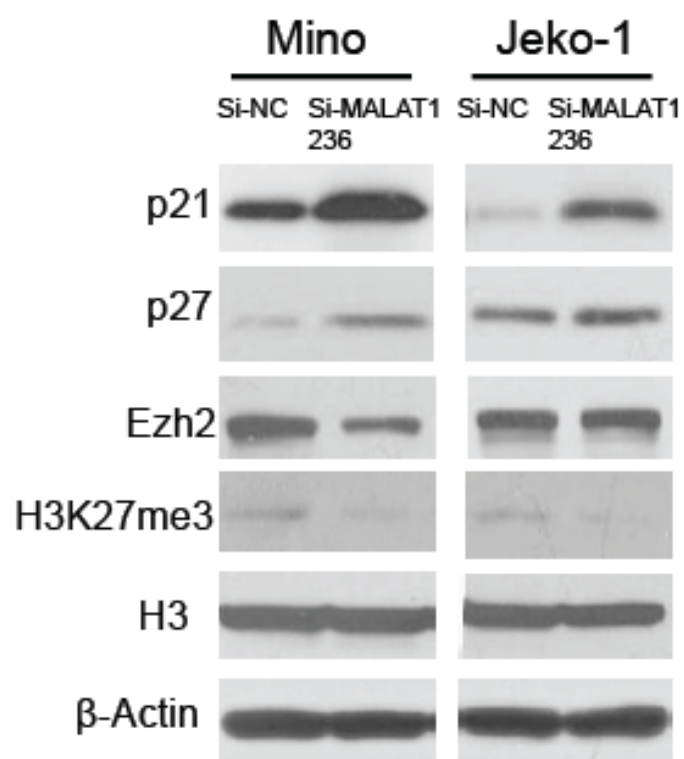

Supplement: Supplementary file 10 — Additional file 10: Figure S8. Effect of MALAT1 knocking down on the expression of EZH2 and H3k27me3, and cell cycle regulators p21 and p27. The expressions of EZH2 and H3k27me3 were moderately suppressed, while p21 and p27 were increased in MALAT1 knock down cells (Mino and Jeko-1) as analyzed by Western blot. [file 12967_2016_1100_MOESM10_ESM.pdf]
