# Supplementary material for: LncRNA MALAT1 promotes development of mantle cell lymphoma by associating with EZH2
Source: J Transl Med. 2016 Dec 20;14:346. doi: 10.1186/s12967-016-1100-9 (PMC5175387; doi:10.1186/s12967-016-1100-9)

Additional file **5**: Figure S4

%	Mino si-NC	Mino si-Malat1	Jeko-1 si-NC	Jeko-1 si-Malat1
Early Apoptosis	2.35	4.49	0.72	1.32
Late Apoptosis	7.89	13.99	6.36	15.98
Living Cells	88.85	78.72	92.66	82.31
Necrosis	0.90	2.80	0.29	0.38

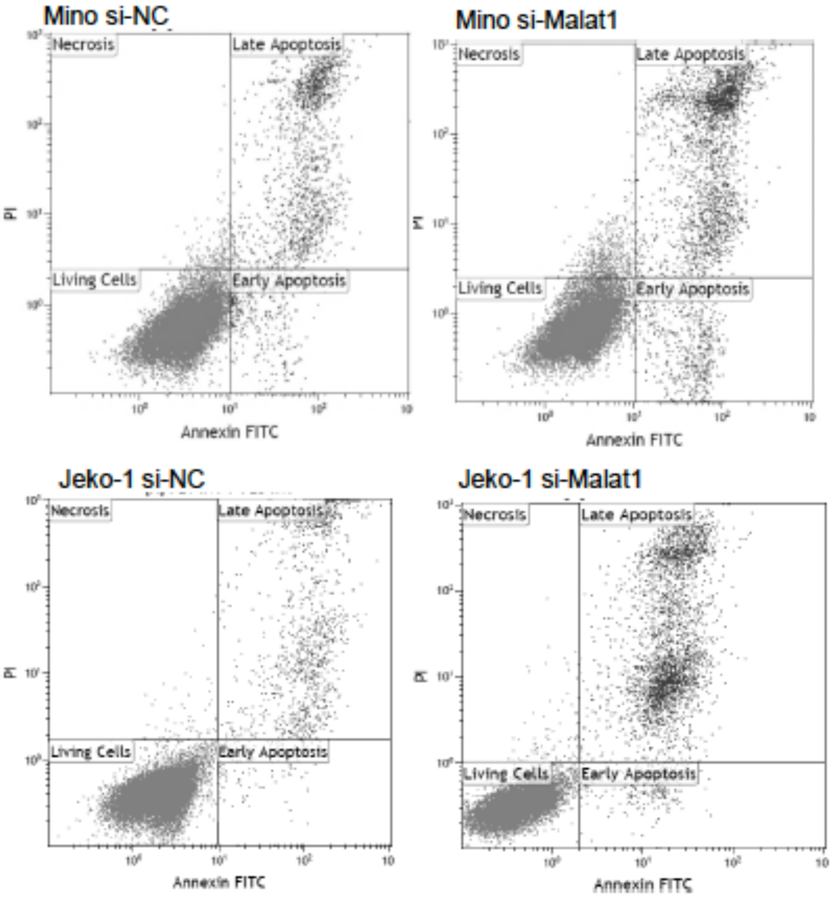

Supplement: Supplementary file 5 — Additional file 5: Figure S4. MALAT1 knockdown enhances MCL cell apoptosis in vitro. In flow cytometric analysis of annexin V/PI staining, the percentage of either early or late apoptotic cells increases in MALAT1 knock down cells. [file 12967_2016_1100_MOESM5_ESM.pdf]
