# Supplementary material for: LncRNA MALAT1 promotes development of mantle cell lymphoma by associating with EZH2
Source: J Transl Med. 2016 Dec 20;14:346. doi: 10.1186/s12967-016-1100-9 (PMC5175387; doi:10.1186/s12967-016-1100-9)
Supplement: Supplementary file 9 — Additional file 9: Table S2. Association of EZH2 expression with clinical parameters of MCL patients. [file 12967_2016_1100_MOESM9_ESM.docx]

Additional file 9: Table S2 - Association of EZH2 expression with clinical parameters of MCL patients.

|  |  | Total  n(%) | EZH2 Low  n(%) | EZH2 High  n(%) | Chi Square test  P value |
| --- | --- | --- | --- | --- | --- |
| Age* | <60y | 11(27.5) | 7(17.5%) | 4(10%) | 0．288 |
|  | ≥60y | 29(72.5%) | 13(32.5%) | 16(40%) |  |
| gender | Male | 31(77.5%) | 16(40%) | 15(37.5%) | 0.705 |
|  | Female | 9(22.5%) | 4(10%) | 5(12.5%) |  |
| Risk group^§^ | low | 18(45%) | 15(37.5%) | 3(7.5%) | 0.0007 |
|  | intermediate | 12（30%） | 3(7.5%) | 9(22.5%) |  |
|  | high | 10（25%） | 2(5%) | 8(20%) |  |
| Overall survival | Alive | 26（65%） | 16(40%) | 10(25%) | 0.047 |
|  | Dead | 14（35%） | 4(10%) | 10(25%) |  |

*Median age: y (range): 62.9(43-80)

§Risk group by MIPI score: high-risk group (score≥6.2), intermediate risk group (5.7≤score<6.2), low-risk group (score<5.7)
